# Continuous Flow Processes as an Enabling Tool for the Synthesis of Constrained Pseudopeptidic Macrocycles

**DOI:** 10.1021/acs.joc.1c03081

**Published:** 2022-02-15

**Authors:** Ferran Esteve, Raul Porcar, Santiago V. Luis, Belen Altava, Eduardo García-Verdugo

**Affiliations:** † Departamento de Química Inorgánica y Orgánica, Universitat Jaume I, Av. Sos Baynat s/n, 12071 Castellón, Spain; ‡ Departamento de Química Orgánica y Bio-orgánica, Facultad de Ciencias, Universidad Nacional de Educación a Distancia, UNED, Avda. Esparta, 28232 Las Rozas, Madrid, Spain

## Abstract

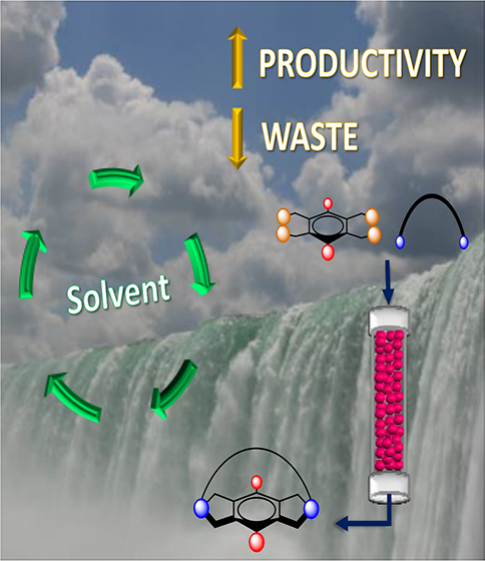

Herein we report our efforts to develop a continuous flow methodology for the efficient preparation of pseudopeptidic macrocyclic compounds containing the hexahydropyrrolo-[3,4-*f*]-isoindolocyclophane scaffold and involving four coupled substitution reactions in the macrocyclization process. Appropriate design of a supported base permitted the continuous production of the macrocycles even at large scales, taking advantage of the positive template effect promoted by the bromide anions. In addition, the use of flow protocols allowed a ca. 20-fold increase in productivity as well as reducing the environmental impact almost 2 orders of magnitude, in comparison with the related batch macrocyclization process.

## Introduction

Success in the synthesis of macrocyclic structures with good yields involves a delicate balance of kinetic and thermodynamic factors.^1^ The most usual approach to the preparation of macrocyclic structures is based on kinetically favoring intramolecular versus intermolecular reactions. According to their different kinetic laws, this can be achieved by modifying the relative concentrations of the active species. Thus, the use of high-dilution conditions is often required to favor the macrocyclization process.^2^

In some cases, the prevalence of an appropriate conformation of the immediate precursor, providing a favorable preorganization for the macrocyclization step, can be an essential factor for the process to be high yielding.^3^ Alternatively, the proper conformation can be induced by the presence of a second species (template).^4^ A supramolecular preorganization event, as induced by a template, incorporates molecular information from the guest to the formed supramolecular species. But even in systems and approaches where the structural or induced conformations of the open-chain precursors favor the macrocyclization reaction, the effect of reaction conditions are still critical for success.

The well-documented application of small-scale flow reactors has often proved to offer considerable advantages over batch reactor designs in synthetic processes.^5^ Continuous flow reactions permit very efficient heat transfer and, therefore, good control of the reaction temperature, avoiding the problems associated with highly exothermic reactions. Mass transfer is also enhanced due to the lower reaction volume and increased contact area, and the use of dangerous or air- and moisture-sensitive compounds can be facilitated by the sealed reactors.^6^ Optimization of reaction conditions is eased by an appropriate control of the residence time, and the scale-up can be achieved by simply pumping, mixing, and quenching the reagents continuously through the reactor for longer periods of time.^7^ Thus, this approach allows a shortening of the time from research to development and production.^8^

Macrocyclization processes under continuous flow conditions is a seldom explored field of research. There are, however, a few precedents in the literature highlighting the opportunities provided by continuous flow processes for the synthesis of macrocycles.^9^ In general, the macrocyclizations considered took place through either intermolecular or intramolecular bond formation enabled by transition metal catalysts. Hence, carbon–carbon bond forming macrocyclizations have been achieved by a Glaser–Hay coupling,^10^ or by an azide–acetylene cycloaddition reaction at high temperature, which did not proceed under batch conditions.^11^ In the case of intermolecular macrocyclizations, different chiral and achiral pyridino-18-crown-6 ethers were synthesized by alkylation of various alcohols in the presence of pyridine diiodides in a continuous-flow Williamson type synthesis using KOH as the base in a packed bed reactor. This provided a cheaper and less dangerous method with higher yields and in shorter times than the ones reported using NaH as a base in batch conditions.^12^ In a similar manner, the use of a K_2_CO_3_ packed bed reactor enabled the preparation in high yields and high purities of C_2_-symmetric chiral PyBox. The macrocyclization reaction relied on amide formation of chlorinated chelidamic acids in the presence of different amino alcohols, and the results obtained by flow processes were not attainable under batch conditions.^13^ Continuous flow methodologies have also been applied for the preparation of cage molecules via a 3-fold homocoupling macrocyclization reaction or by dynamic covalent imine forming reactions.^14,15^

These reduced number of precedents suggest that continuous flow methodologies pave the way to develop more efficient macrocyclization reactions. The success of this underexploited approach is based on the precise control of reagent concentration via static mixers, the use of supported reagents and catalysts, and the superior heat and mass transfer only achievable under flow conditions. As such our efforts have been devoted to developing a continuous flow methodology for the preparation of constrained macrocyclic pseudopeptides.

## Results and Discussion

We have recently reported a highly selective anion-templated synthesis of a conformationally constrained pseudopeptidic macrocyclic cyclophane (**3a**).^16^ The optimal preorganization of the reagents and the intermediate, together with the positive template effect of the bromide anion, provided an efficient macrocyclization reaction with excellent yield and selectivity (higher than 95%) in CH_3_CN using Cs_2_CO_3_ as the base. This macrocyclic system can act as an efficient organocatalyst for the conversion of CO_2_ under relatively mild conditions.^17^ In an attempt to develop new structures of this family, the synthesis of **3b** was attempted under batch conditions, although unsuccessfully. Most likely, the lower macrocyclization efficiency attained when using **1b** is the result of the steric hindrance provided by the additional groups on the aromatic scaffold. In the search of alternative approaches and to provide an easier scale-up for the synthesis of these pseudopeptidic macrocyclic cyclophanes (**3**), the use of flow chemistry was considered.

**Scheme 1:**
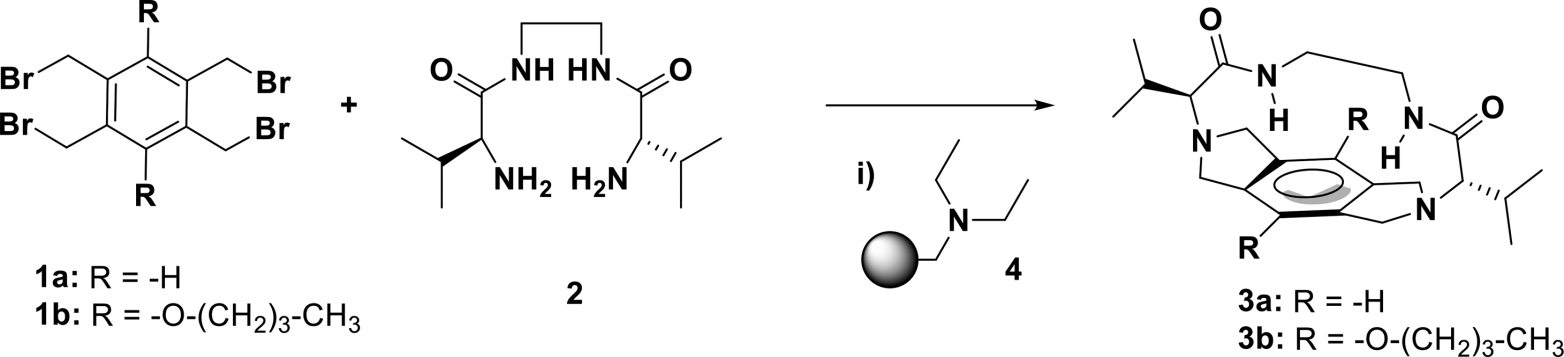
Macrocyclization Reaction between **1a**,**b** and **2** Conditions: (i) 3 h, 80 °C, 6 equiv of base in CH_3_CN, 2 mM.

Although the use of packed bed reactors using inorganic bases has been formerly reported,^12,13^ the partial solubility of Cs_2_CO_3_ in CH_3_CN hampered this approximation, as some leaching could not be avoided. Thus, to develop this macrocyclization under continuous-flow, the design of an appropriate immobilized base to substitute the Cs_2_CO_3_ was envisaged. Among the different solid supported bases,^18^ the polymer immobilized tertiary amine **4** was selected as it was easily obtained by modification of a Merrifield resin (chloromethyl polystyrene-divinylbenzene macroporous resin, 5.5 mmol Cl/g nominal loading) with diethylamine (see Scheme S1 and Figures S11 and S12 for the synthesis and characterization of **4**). The efficiency of this supported base was initially evaluated in the macrocyclization reaction between **2** and **1a** under batch conditions (Scheme 1) in the presence of **4** instead of Cs_2_CO_3_ (6 equiv in both cases).

After 3 h of reaction, the crude was filtered off and the solvent was removed under a vacuum, affording pure **3a** in 82% isolated yield. The supported base was straightforwardly recovered from the reaction crude and analyzed by IR spectroscopy. The FT-IR-ATR spectrum of the used base showed the appearance of a broad band at 2800–2400 cm^–1^ corresponding to the formation of ammonium salts (Figure S1).^19^ The reactivation of the protonated resin could be attained by simply washing the resin with a 0.5 M KOH methanolic solution, observing the disappearance of the ammonium broad bands in the IR spectrum (Figure S1). The base could be used again in the macrocyclization reaction for 15 runs without any decrease in the efficiency. These initial results support the use of **4** as suitable replacement of Cs_2_CO_3_. Indeed, when the kinetic profiles of the reaction in the presence of **4** or Cs_2_CO_3_ were compared, a slightly faster profile was obtained using **4** instead of Cs_2_CO_3_, reaching NMR yields higher than 90% in 2 h (Figure S2).

**Figure 1:**
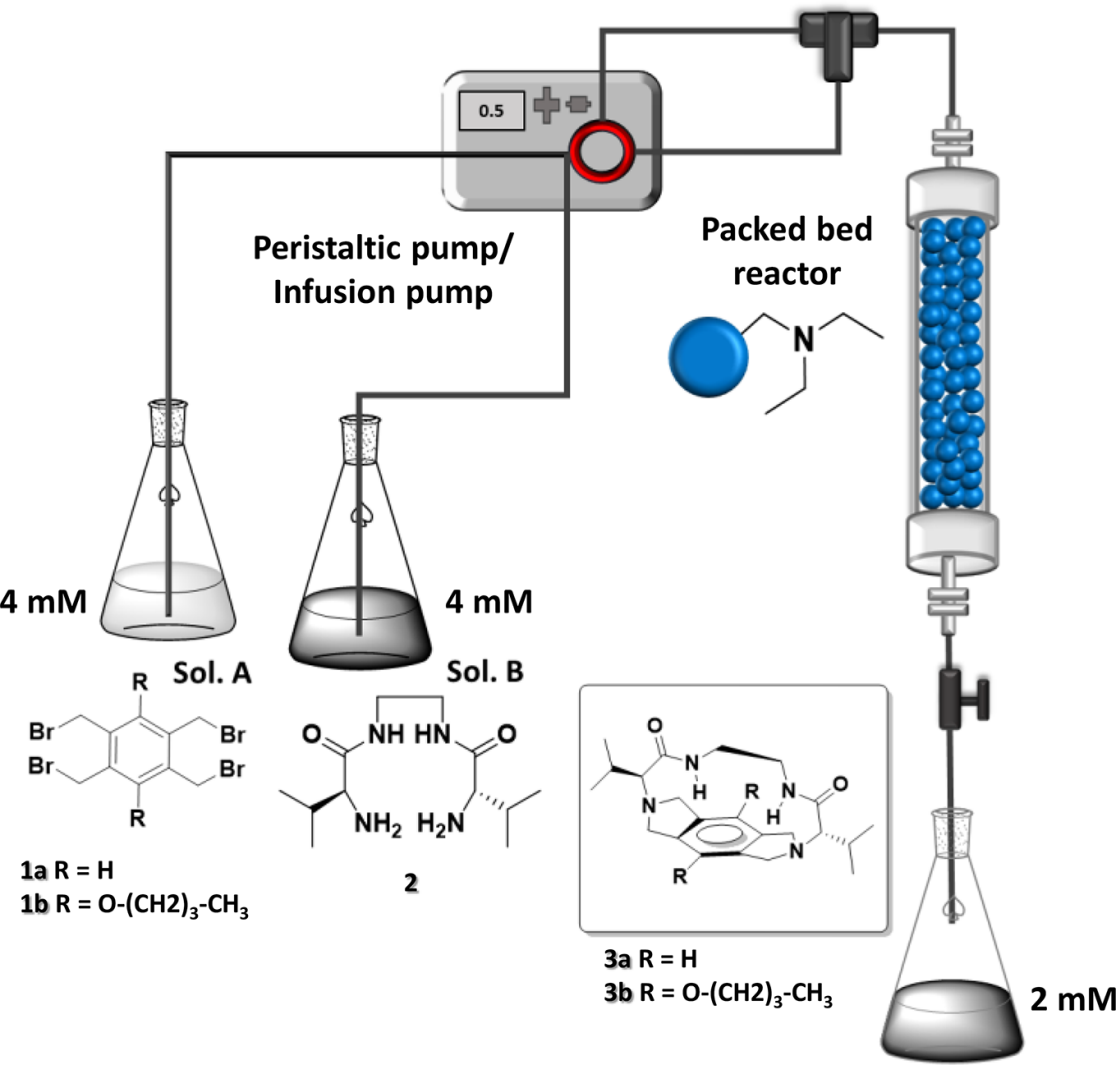
Schematic representation of the flow setup for the continuous flow macrocyclization. Solution A: **1**, 4 mM in CH_3_CN. Solution B: **2**, 4 mM in CH_3_CN. Infusion pumps were generally used for the experiments. Peristaltic pumps were used for scale-up experiments.

Once the efficiency of **4** as a supported base was proved for the macrocyclization under batch conditions, its behavior under continuous-flow conditions was evaluated. The experimental setup is shown in Figure 1 (see also Figure S15). The two reagents (4 mM solutions in CH_3_CN) were pumped separately (solutions A and B) and mixed before entering the packed bed reactor. Noteworthy, if both reagents were mixed before pumping, part of the initial bisaminoamide **2** acted as the base. This led to the formation of the desired macrocycle but also to the precipitation of corresponding dishydrobromide salt of **2** with the blockage of the flow reactor. It should also be pointed out that a higher concentration of the reagents reduced the efficacy of the macrocyclization and led to precipitation of the final macrocycle. Thus, the final concentration at the mixing point was fixed to 2 mM in CH_3_CN.

The reaction mixture was pumped through a packed bed reactor loaded with the supported base **4** (4.7 mmol amine/g) at different flow rates while heated at 80 °C. Table 1 illustrates the results obtained. The use of a 1000 μL/min flow rate (entry 1, Table 1) led to promising NMR yields of 69% with only 2.1 min of residence time, demonstrating the suitability of the reaction setup to convert the reagents into the desired macrocycle. The increase in residence time from 2.1 to 10.7 min by reducing the total flow rate from 1000 to 400 μL/min (entries 1 to 3, Table 1) resulted in progressive increments of the NMR yields of the desired [1 + 1] cyclic pseudopeptide **3a**. To our delight, a 99% NMR yield was attained for a total flow of 200 μL/min using a reactor of only 2.16 mL (entry 4 in Table 1). This result is outstanding as under batch conditions, reaction times of ca. 2 h are required to achieve a similar yield.

**Table 1:** Optimization for the Macrocyclization between **1a** and **2** under Flow Conditions^a^

entry	total flow (μL/min)^b^	residence time (min)	yield (%)^c^
1	1000	2.1	69
2	700	3.1	79
3	400	5.4	91
4	200	10.7	99

a All the experiments were carried out for 1 g of basic resin **4**. Column volume: 2.16 mL.

b Total flow rate corresponds to equal flow rates of each reagent to maintain a ratio of 1:1.

c ^1^H NMR yields. Yields calculated for aliquots taken after pumping a volume of reagents equal to at least three times the packed void volume of the reactor.

**Figure 2:**
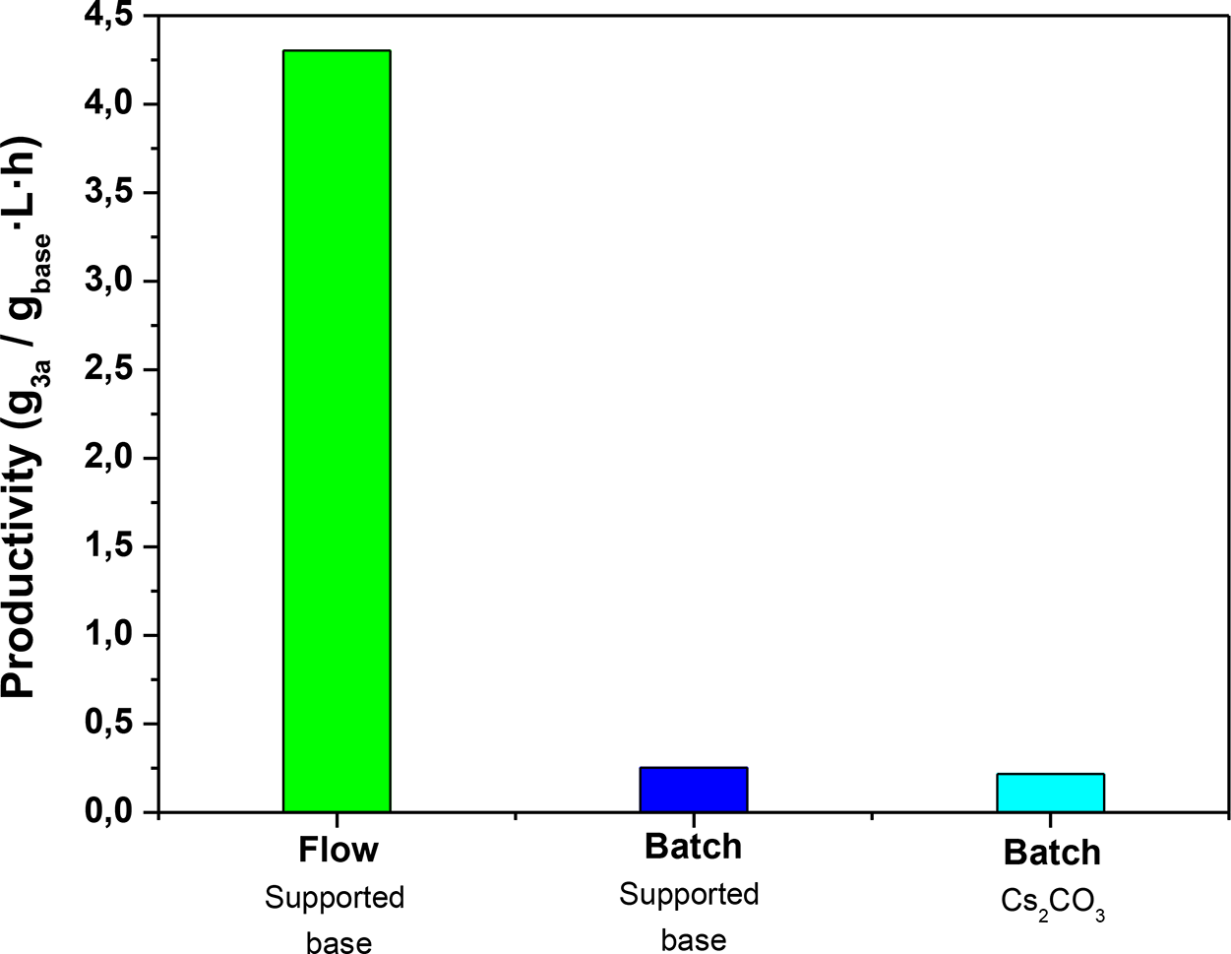
Productivities obtained using acetonitrile as solvent (2 mM final concentrations of **1a** and **2**) at 80 °C. Batch conditions: cesium carbonate (light blue), **4** (blue); flow conditions with **4** (green). Reaction times: 10.7 min for flow conditions, 2.5 h for batch conditions using **4**, and 3 h for batch conditions using Cs_2_CO_3_.

To compare the macrocyclization under batch and flow conditions, the volumetric productivity per gram of base for both systems was calculated considering the yields of the isolated product (see Experimental Section for details). Remarkably, comparing the productivity of the flow-based process (4.303 g_**3a**_/g_base_·L·h) with the ones obtained in batch conditions using either Cs_2_CO_3_ (0.216 g_**3a**_/g_base_·L·h) or the supported base **4** (0.252 g_**3a**_/g_base_·L·h) indicated an almost 20-time increase on productivity in favor of the flow process (Figure 2).

This enhancement on the reaction productivity is likely to be related with the higher local concentration of the base in the flow setup. For instance, in the reactor of 2.16 mL, at a flow rate of 200 μL/min, an instant ratio of 1300 mol of base/mol of **2** can be achieved, while in the batch process the ratio was fixed to 6 mol of base/mol of **2**.

A second possible effect can be attributed to the formation of the corresponding supported salt bromide (R_3_NHBr) during the process. This salt can play a double role, as previously demonstrated for this macrocyclization.^16^ The bromide anion acts as an efficient template facilitating the [1 + 1] macrocyclization process. The strong intramolecular associations of the open-chain intermediate through NH_amide_···Br^–^···NH_amide_ hydrogen bonds provide its appropriate folding favoring macrocyclization and thus hampering oligomerization reactions. Additionally, the bromide salt can enhance the kinetics of the macrocyclization by increasing the nucleophilic character of the terminal amino groups of **2**.

**Figure 3:**
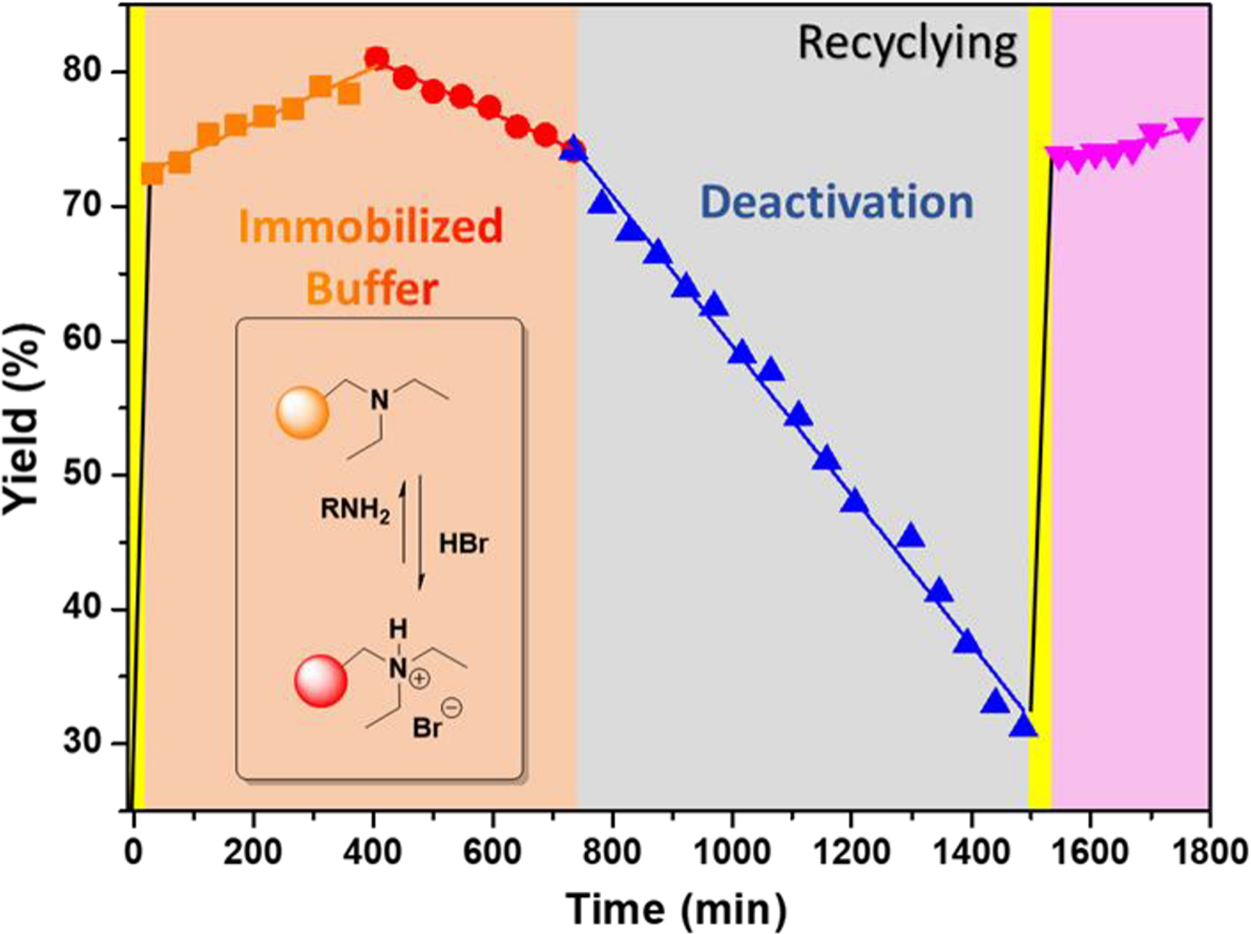
Macrocyclization yield of **3a** vs TOS. The ranges marked in yellow correspond to the loading of the reagents in the reactor and the reactivation process. The area highlighted in orange is assigned to the regime in which concentration of the NR_3_/R_3_NHBr supported species are high enough. Time ranges in gray correspond to the deactivation of the supported base. The time range marked in pink corresponds to the macrocyclization after the reactivation of the basic resin, being comparable to the situation in the first (orange) stage. Conditions: 400 μL/min (2 mM) for 2.2 min. Yields for **3a** were determined by ^1^H NMR.

To analyze the effects of the supported base and its conjugated acid formed during the reaction, the macrocyclization process was studied at moderate yields of **3a**. Figure 3 shows the evolution of reaction profile in terms of yield of **3a** vs the time on stream (TOS) obtained by pumping the reagents (total flow = 400 μL/min) through a packed bed reactor of 0.87 mL (packed void volume; residence time = 2.2 min).

The results showed that at early times on stream the reaction achieves an NMR yield of ca. 70%. The high excess of the base in comparison with the pseudopeptide **2** is likely to contribute to this good yield in a short contact time (2.2 min). As the time on stream increases, the basic supported reagent is consumed and transformed into its corresponding conjugated acid. Thus, a steady decrease in activity after a certain time on stream should be expected. However, a significant increase on the NMR yield was observed during the first 7 h of continuous production of macrocycle **3a**, rendering a maximum value of 81% after 450 min on the stream. This initial steady enhancement of the NMR yield with time can be attributed to the generation of the ammonium bromide salts, triggering the template-assisted macrocyclization.^16^ It must be noted that this maximum represents the optimal ratio between the amount of base needed for the process to occur efficiently and the amount of bromide template favoring the macrocyclization. After this point, the amount of ammonium bromide continues increasing with the concomitant decrease in the amount of available base. This leads to a decrease in NMR yield. Such a decrease is initially slow, between 450 and 725 min, showing a slope similar but with opposite sign to the one observed from 0 to 450 min. Overall, during this first stage (0–725 min, orange in Figure 3) the system is quite efficient as the concentration of both participant species, the base and the bromide salt, are kept sufficiently high as compared to that of the reagents pumped.

After 750 min on stream, the concentration of the basic sites onto the support becomes too small, and the NMR yields suffer a more drastic decrease with time until reaching a value of only ca. 35% after 1500 min. At this point, the amount of reagents fed to the reactor corresponded to the theoretical value of base loaded.

At this time, the flow of the reagents was stopped and 30 mL of a 0.5 M solution of KOH in methanol was pumped through the reactor at 1000 μL/min, allowing to fully recover the supported base (as previously corroborated in Figure S1). When the pumping of the reagents was restarted, similar yields to those initially observed were obtained (pink region in Figure 3).

In the view of these results, and to prove the positive effect produced by the in situ generation of the bromide ammonium salt, a packed bed reactor was loaded with a polymer cocktail,^20^ containing an equimolar mixture of supported amine (**4**) and an analogous supported bromide salt (**5**, Scheme S1). The macrocyclization was carried out under the same conditions reported above. The efficiency of the system, in terms of productivity, for the initial 150 min was constant and as high as the best obtained for the initial setup using only the supported base **4**. After a certain period, the productivity of the system decayed in a similar way to that previously observed for the supported base **4** (Figure S3). These results confirmed that the presence of the supported bromide enhanced the efficiency of the macrocyclization reaction.

**Scheme 2:**
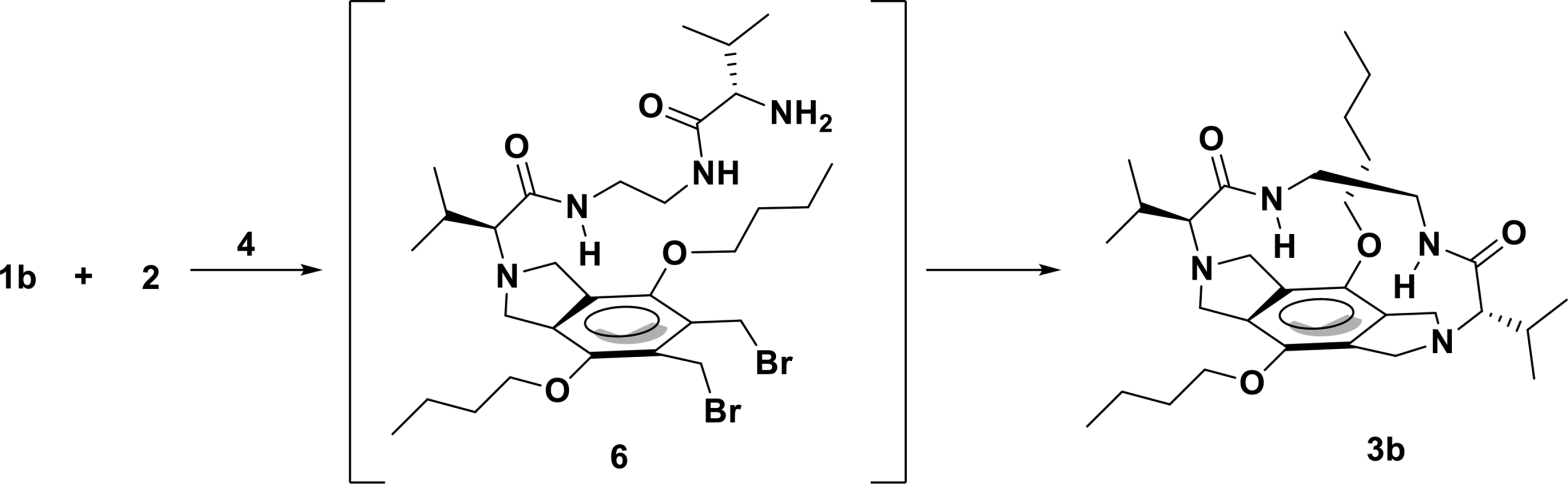
Macrocyclization Reaction between **1b** and **2** Conditions: 3 h, 80 °C, 6 equiv of **4** in CH_3_CN, 2 mM in **1b** and **2**.

Encouraged by these improvements, the continuous flow synthesis of an analogous pseudopeptidic macrocycle **3b** was also evaluated. The macrocyclization reaction between **2** and **1b** was more challenging due to the electronic and steric factors introduced by the presence of the butoxy groups. Compound **1b** was obtained as reported in the literature with a 22% yield (Scheme S2).^21^ Indeed, under batch conditions, even when the macrocycle **3b** could be isolated and fully characterized by spectroscopic techniques, only a poor isolated yield (3% of **3b**) was obtained. The lower macrocyclic efficiency can be attributed to the poorer preorganization of the reaction intermediate **6** in which only one of the terminal amino groups of **2** had reacted with two adjacent bromomethyl groups of **1b** (Scheme 2).

**Figure 4:**
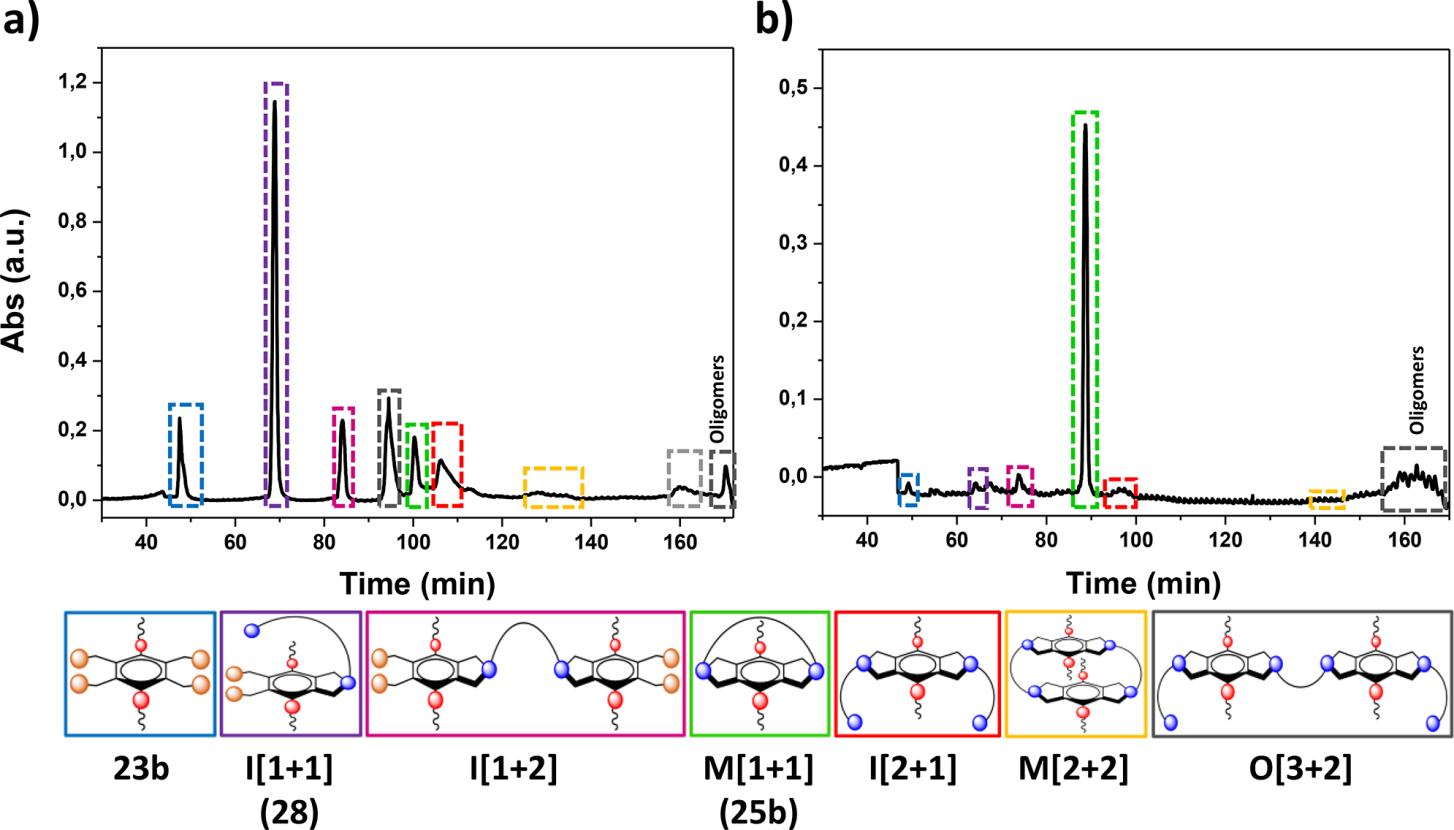
Flash chromatograms (UV-wavelength: 220 nm) obtained for the purification of the crude of the macrocyclization reaction between **2** and **1b**, using CH_2_Cl_2_ and MeOH as the eluents. The predominant component of each fraction has been highlighted. Peaks in green correspond to the desired [1 + 1] macrocycle **3b**. (a) Batch conditions. (b) Continuous flow system.

This favors the intermolecular reactions instead of the intramolecular process, resulting in a wide range of oligomeric/polymeric side products detected by MS, NMR, and flash chromatography (Figure 4 and Figure S4) with a poor selectivity toward the desired macrocycle (Table 2). Clear evidence of the reduced macrocyclization efficiency for **3b** was the detection and isolation in a ca. 5% yield of the open-chain [1 + 1] intermediate **6** (Scheme 2). In the case of the cyclization between **1a** and **2**, the analogous intermediate could not be detected nor isolated, as the preorganization of the reagents and the template and catalytic effect of the bromide permitted the four substitution reactions to take place almost simultaneously.^16^

**Table 2:** Comparison of the Product Distribution Obtained under Batch and Flow Conditions for the Reaction between **1b** and **2**^a^

		product distribution (%)^b^	
entry	product^a^	batch	flow
1	I [1 + 1] (**6**)	5	1
2	I [1 + 2]	4	2
3	M [1 + 1] (**3b**)	5	20
4	I [2 + 1]	19	3
5	M [2 + 2]	7	2
6	O [3 + 2]	8	2
7	Oligomers/Polymers	42	60
8	Others	10	10

a I = intermediate, M = macrocycle, O = oligomer. Numbers in brackets [*n* + *m*] correspond to the stoichiometric factor for **2** (*n*) and **1b** (*m*). See Figure 4 for the identification of the different species.

b Product distribution calculated after separation and purification by flash chromatogram using CH_2_Cl_2_ and MeOH as the eluents. Product distribution for the impure fractions has been calculated as their most abundant component identified by ESI-MS(+) and/or ^1^H NMR. Batch conditions: 3 h, 80 °C, 6 equiv of **4** in CH_3_CN, 2 mM in **1b** and **2**. Flow conditions: total flow 200 μL/min, 80 °C, CH_3_CN (2 mM final concentration), residence time: 10.7 min

When the reaction was performed following the same optimization protocol described for the synthesis of **3a** under flow conditions, using **4** as supported base, a significant increase in the conversion of **1b** was observed, as compared with the one attained under batch conditions (Figure S5). In fact, conversions above 95% were obtained using a flow rate of 200 μL/min, corresponding with a residence time of 10.7 min, while the conversion under batch conditions was only 70% after 1 h. This remarkable acceleration of the reaction led to a reduced number of side products (Figure 4 and Table 2). Again, the higher actual concentration of base and the presence of the bromide anions must contribute to this improved performance. The 19% isolated yield obtained for **3b**, under flow conditions, represents a ca. 6-time improvement in comparison with the yield obtained under batch conditions (3%). This led to remarkable higher productivities. Whereas the productivity under batch conditions for the synthesis of **3b** using **4** as supported base was 0.014 g_**3b**_/g_base_·L·h, it could be increased up to 1.126 g_**3b**_/g_base_·L·h with the continuous flow methodology, corresponding to a ca. 80-time increase. These results highlight how the continuous flow processes can be exploited for enhancing the yield, selectivity, and productivity of the macrocyclization reactions in comparison with the reaction under batch conditions.

The continuous flow process also provided significant advantages for the simple production of the macrocycle at the gram scale. When a 3-time scale-up of the macrocyclization under batch conditions was assayed, a reduction of the isolated yield of **3a** from 85% to 43% was observed. However, under flow conditions, using a reactor of 12.4 mL loaded with 6 g of the supported base **4** and a total flow (provided by a peristaltic pump) of 1160 μL/min for 17 h, it was possible to synthesize up to 0.891 g of **3a**, corresponding with a 98% isolated yield (see Experimental Section for additional details).

**Figure 5:**
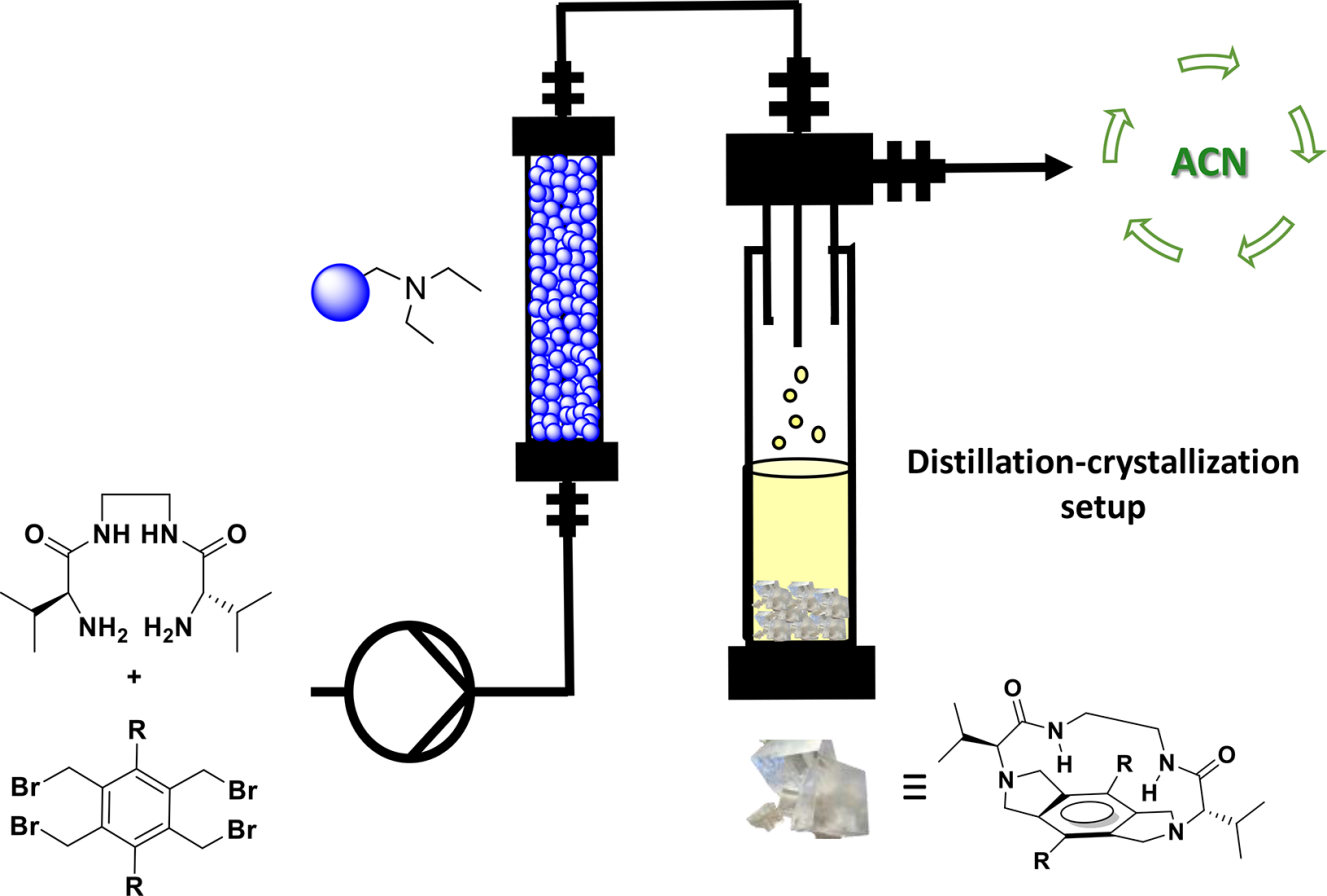
Integration into the flow process of a distillation-crystallization setup for the synthesis of constrained pseudopeptidic macrocycles. The amount of acetonitrile recovered (up to 85%) was used to form the subsequent reagent solutions.

The continuous flow macrocyclization process enabled by the supported base opens the possibility to integrate, in a single process, the synthesis of the macrocycle, its purification, and the separation and reuse of the solvent used in excess. This integration may contribute to increase the sustainability of the process even when high-dilution conditions are used. With this idea in mind, the continuous flow system was coupled with a distillation setup (Figure 5).

This simple setup allowed to obtain a stationary stream of high purity recovered solvent (Figure S6). The average distillation flow of the distillation stream was of 198 μL/min, in good agreement with the macrocyclization flow input (200 μL/min). The total recovery over the time on stream reached up to 85% as some solvent remained on the distillation device. Additionally, the continuous distillation of the solvent afforded a continuous increase in the concentration of the macrocycle in the final solution. This resulted in the formation of crystals in the distillation flask (Figures S7 and S8). The chemical composition of the crystals was determined by NMR and IR analyzes, indicating that the only product formed was **3a** with a 69% isolated yield (Figure S9).

With this novel approach, a significant reduction of the E-Factor was accomplished (Table 3).^22^ For instance, a decrease of 1 order of magnitude for the E-Factor was observed when comparing the batch and flow syntheses of **3a** (see entries 1–3 in Table 3). In a similar trend, the coupled flow synthesis-isolation for **3b** resulted in a much lower environmental impact for the macrocyclization (entries 5 and 6, Table 3). Comparing entries 1, 4, and 5 (Table 3), one may appreciate the drastic environmental implications related with chromatographic protocols. These results highlight the paramount role of designing novel techniques for increasing the macrocyclization efficiency and selectivity.

**Table 3:** Summary of the Macrocyclization Efficiencies in Terms of Productivity and E-Factor Obtained for Macrocycles **3a** and **3b**

entry	comp.	method	yield (%)	productivity (g_p_/L·h)	E-Factor
1	**3a**	Batch	82	0.252	1033
2		Flow	98	4.303	1030
3		Flow^a^	69	2.977	155
4	**3b**	Batch	3	0.014	371 209
5		Batch	3	0.014	1033^b^
6		Flow^a^	19	1.126	155^b^

a Including reaction, purification or crystallization, and solvent recovery.

b The waste generated during the chromatographic purification has not been considered in the waste calculation. Reaction conditions: 80 °C, 6 equiv of **4** in CH_3_CN, 2 mM in **1b** and **2**.

## Conclusions

The present results highlight that conducting macrocyclizations in continuous flow can offer several important advantages. This has been illustrated for the flow syntheses of constrained pseudopeptidic macrocycles. The higher productivity and lower environmental impact obtained under flow conditions can be correlated with the more efficient template effect within the packed bed reactor, and with the integration of the isolation and solvent reuse in a simple setup. To the best of our knowledge, this is the first system reported up to date integrating macrocyclization, crystallization with high quality crystals, and solvent recovery under flow conditions. Besides, these continuous-flow macrocyclization can be easily scaled-up by simply pumping the reaction mixture continuously through the reactor for a given period of time.

## Experimental Section

### General Methods

NMR experiments were carried out at 400 or 300 MHz for ^1^H and 100 or 75 MHz for ^13^C. Chemical shifts are reported in ppm from tetramethylsilane using the solvent resonance as the internal standard. FT-IR spectra were recorded using an ATR adapter. HRMS were recorded with a Q-TOF instrument. Optical rotation was determined with a digital polarimeter (Na: 589 nm). Melting points were measured using a standard apparatus and are uncorrected. Unless otherwise stated, all reagents were commercially available and not purified before use. The pseudopeptidic reagent **2** was prepared following literature procedures.^23^

### General Procedure for Synthesis of Compound **1b**

The aromatic reagent **1b** was obtained according to literature procedures (Scheme S2).^21^ The overall yield for the reaction was 22% (3.765 g). Characterization: IR (ATR) 2960, 1434, 1274, 1020, 632 cm^–1^; ^1^H NMR (400 MHz, CDCl_3_) δ 4.76 (s, 8H), 4.16 (t, *J* = 6.6 Hz, 4H), 1.89–1.94 (m, 4H), 1.58–1.62 (m, 4H), 1.05 (t, *J* = 7.4 Hz, 6H); ^13^C{^1^H} NMR (100 MHz, CDCl_3_) δ 153.8, 133.4, 75.1, 32.5, 23.4, 19.3, 14.1. The structure of the compound was confirmed by single crystal X-ray diffraction (Figure S10 and Table S1). CCDC number: 2084454.

### General Procedure for Synthesis of Compound **4**

The macroporous Merrifield resin (5.00 g, 5.5 mmol Cl/g, 27.5 mmol of Cl) was introduced in a round-bottom flask (50 mL). DMF (10 mL) and diethylamine (17.1 mL, 165 mmol) were then added, and the mixture was heated at 80 °C on a heating mantle with gentle stirring overnight. The reaction crude was filtered off under a vacuum and the resin was washed with DMF (3×), MeOH (3×), and CH_2_Cl_2_ (3×). After vacuum drying at 50 °C for 5 h, 5.71 g of resin were obtained (95%, 26.1 mmol of amine). Characterization: absence of C–Cl band (1264 cm^–1^) in the FT-IR spectrum (Figure S11); the full absence of C–Cl bonds was also confirmed by a negative NBP test (Figure S12);^24^ Calcd %N for full conversion 6.4%; Exp. 6.6%. TGA decomposition temperature >350 °C.

### General Procedure for Synthesis of Compound **5**

Resin **4** (1.5 g, 6.9 mmol) was introduced in a round-bottom flask (25 mL). DMF (4 mL) and *n*-bromobutane (3.0 mL, 27.6 mmol) were added and the mixture was heated at 80 °C on a heating mantle with gentle stirring overnight. The reaction crude was filtered off under a vacuum and the resin was washed with DMF (3×), MeOH (3×), and CH_2_Cl_2_ (3×). After vacuum drying at 50 °C for 12 h, 2.74 g of resin were obtained (97%, 26.1 mmol of ammonium salt). Characterization: appearance of a broad band at 2800–2200 cm^–1^, representative of ammonium salts (Figure S13); negative NBP test (Figure S14);^24^ Calcd %N for full conversion 4.1%, Exp. 4.4%. TGA decomposition temperature >200 °C.

### Batch Syntheses

#### Synthesis of **3a** Using Cs_2_CO_3_ as the Base

Pseudopeptide **2** (132 mg, 0.512 mmol) and **1a** (242 mg, 0.512 mmol) were dissolved in acetonitrile (255 mL), and Cs_2_CO_3_ (1000 mg, 3.069 mmol, 6 equiv) was added. The reaction mixture was refluxed on a heating mantle with magnetic stirring for 3 h, and then the solvent was evaporated under a vacuum. The resulting residue was treated with distilled water, centrifuging the final suspension at 3000 rpm for 8 min to afford pure **3a** as a solid. Yield: 167 mg (0.434 mmol, 85%). For complete characterization see ref (16).

#### Synthesis of **3a** Using **4** as the Base

Pseudopeptide **2** (197 mg, 0.765 mmol) and **1a** (362 mg, 0.765 mmol) were dissolved in acetonitrile (383 mL), and the supported base **4** (1000 mg, 4.588 mmol, 6 equiv) was added. The reaction mixture was refluxed on a heating mantle with gentle magnetic stirring for 2.5 h, and then the basic resin was filtered off. The resulting residue was evaporated under a vacuum to yield pure **3a** as a yellowish solid. Yield: 241 mg (0.627 mmol, 82%).

#### Synthesis of **3b** Using Cs_2_CO_3_ as the Base

Pseudopeptide **2** (132 mg, 0.512 mmol) and **1b** (304 mg, 0.512 mmol) were dissolved in acetonitrile (255 mL), and Cs_2_CO_3_ (1000 mg, 3.069 mmol, 6 equiv) was added. The reaction mixture was refluxed on a heating mantle with magnetic stirring for 6 h, and then the solvent was evaporated under a vacuum. The resulting residue was treated with distilled water, centrifuging the final suspension at 3000 rpm for 8 min, and the resultant solid was purified by flash chromatography using MeOH:CH_2_Cl_2_ as the eluent. Yield: 8 mg (0.015 mmol, 3%). Characterization: [α]_D_^25^ +9.6° (*c* = 0.1, MeOH); IR (ATR) 3310, 1645, 1523, 1290, 1239 cm^–1^; ^1^H NMR (400 MHz, CDCl_3_) δ 5.71 (s, 1H), 4.34 (d, *J* = 15.1 Hz, 1H), 4.21 (d, *J* = 14.5 Hz, 1H), 3.95 (d, *J* = 15.3 Hz, 1H), 3.81–3.91 (m, 3H), 3.24–3.21 (m, 1H) 3.13–3.10 (m, 1H), 2.36 (d, *J* = 10.0 Hz, 1H), 2.06–2.11 (m, 1H), 1.61–1.65 (m, 2H), 1.43–1.45 (m, 2H), 1.09 (d, *J* = 6.7 Hz, 3H), 0.95 (t, *J* = 7.3 Hz, 3H), 0.79 (d, *J* = 6.5 Hz, 3H); ^13^C{^1^H} NMR (100 MHz, CDCl_3_) δ 173.2, 145.9, 134.2, 133.4, 72.6, 59.7, 52.9, 41.1, 32.3, 30.6, 20.0, 19.7, 19.3, 14.0; HRMS (ESI/Q-TOF) *m*/*z* [M + H]+ calcd for C_30_H_48_N_4_O_4_, 529.3754, found 529.3752.

#### Synthesis of **3b** Using **4** as the Base

Pseudopeptide **2** (197 mg, 0.765 mmol) and **1b** (454 mg, 0.765 mmol) were dissolved in acetonitrile (383 mL), and the supported base **4** (1000 mg, 4.588 mmol, 6 equiv) was added. The reaction mixture was refluxed on a heating mantle with gentle magnetic stirring for 6 h, and then the basic resin was filtered off. The resulting residue was evaporated under a vacuum, and the resulting solid was purified by flash chromatography using MeOH:CH_2_Cl_2_ as the eluent (Tables S2 and S3). Yield: 12 mg (0.023 mmol, 3%).

#### Synthesis of **6** Using **4** as the Base

Pseudopeptidic intermediate **6** could be isolated and fully characterized from the chromatographic protocol of the reaction between **2** and **1b**. Characterization: [α]_D_^25^ −6.8° (*c* = 0.1, MeOH); IR (ATR) 3298, 1636, 1542, 1262, 653 cm^–1^; ^1^H NMR (400 MHz, CDCl_3_) δ 7.18 (s, 1H), 4.74–4.83 (m, 2H), 4.15–4.17 (m, 1H), 3.84–3.86 (m, 1H), 3.66–3.72 (m, 2H), 3.35–3.37 (m, 1H), 3.00 (d, *J* = 4.7 Hz, 1H), 1.82–1.84 (m, 2H), 1.53–1.59 (m, 2H), 1.04 (d+t, *J* = 6.6, 5.7 Hz, 6H), 0.93 (d, *J* = 6.8 Hz, 3H); ^13^C{^1^H} NMR (100 MHz, CDCl_3_) δ 173.6, 154.0, 134.5, 132.0, 75.5, 71.8, 46.1, 32.7, 31.7, 23.9, 19.9, 19.4, 18.6, 14.1; HRMS (ESI/Q-TOF) *m*/*z* [M + H]+ calcd for C_30_H_50_Br_2_N_4_O_4_, 689.2277, found 689.2286.

### Kinetic Profiles

Each reaction was performed in a twin-neck round-bottom flask on a heating mantle. Different aliquots were withdrawn from the flask at the desired times and injected in a HPLC instrument in order to determine the conversion of the aromatic reagent **1a** or **1b**, with the appropriate calibrates.

### Flow Equipment

Syringe pumps from kdScientific and Hamilton 25 mL syringes were used. Glass Omnifit columns with variable length and an internal diameter of 0.7854 cm were used as the reactor. For the scale-up experiment, an ISMATEC-REGLO Digital peristaltic pump was used to pump the reagents with a total flow of 1160 μL/min. The reactor could be kept at 80 °C by recirculating water with a Julabo F-83 instrument.

### Flow Optimization

Stock solutions (4 mM) of the different reagents were accurately prepared in 500 mL volumetric flasks. These solutions were separately introduced in the system with two syringe pumps and were mixed before the reactor with a T-shaped piece. The resultant 2 mM reaction mixture was run through an Omnifit (006RG-10-10) column packed with 1000 mg of **4**, resulting in a 2.16 mL basic reactor. This column was immersed in a 500 mL beaker and thermostatted with water at 80 °C (Figure S15). Adjusting the flows provided by the syringe pumps, the effect of the residence time could be elucidated. All the residence times were determined with the breakthrough of compounds. The final solutions were injected in the HPLC instrument to determine the effectiveness of the reaction.

### Flow-Distillation Methodology

A distillation setup was easily connected to the flow system described in the Flow Optimization section. The tubing at the exit of the reactor was introduced into a 100 mL twin-neck round-bottom flask through one of the necks of the flask. A distillation setup was mounted in the other neck of the flask, allowing the solvent to be recovered in a separate flask, and reused for the preparation of new stock solutions (Figure S16). The temperature of the distillation flask on a heating mantle was set at 95 °C, so the distillation temperature was stabilized at 81 °C to yield pure acetonitrile in almost a stationary regime. For **1a**, at the end of the process, crystals of pure **3a** were recovered from the distillation flask. In the case of **1b**, the resultant brownish solid residue of the distillation was purified by flash chromatography to afford pure **3b**.

### Flow Syntheses

#### Flow Synthesis of **3a**

The solutions of **1a** and **2** in acetonitrile (4 mM) were pumped through an Omnifit (006RG-10-10) column packed with 1000 mg of **4** for 10 h. This column was immersed in a 500 mL beaker with water at 80 °C. The residence time was set to 10.7 min by adjusting the total flow to 200 μL/min. While the solvent outcoming from the reactor was being distilled, the formation of crystals could be observed at the bottom of the distillation flask. After recovering and drying off the crystals, pure **3a** was obtained (63 mg, 69% yield, 0.165 mmol). With this system, 85% of the acetonitrile pumped through the system was recovered.

#### Flow Synthesis of **3b**

The solutions of **1b** and **2** in acetonitrile (4 mM) were pumped through an Omnifit (006RG-10-10) column packed with 1000 mg of **4** for 10 h. This column was immersed in a 500 mL beaker with water at 80 °C. The residence time was set to 10.7 min, by adjusting the total flow to 200 μL/min. After 10 h, the resultant brownish solid was purified by flash chromatography, using MeOH:CH_2_Cl_2_ as eluent to yield **3b** as a white solid (Tables S4 and S5). Yield: 24 mg (0.045 mmol, 19%).

#### Scaled-up Flow Synthesis of **3a**

The solutions of **1a** and **2** in acetonitrile (4 mM) were pumped through an Omnifit (006EZ-10-33-AF) column packed with 6000 mg of **4** for 17 h. The reactor was kept at 80 °C by recirculation of water around the reactor (Figure S17). The residence time was set to 10.7 min, by adjusting the total flow to 1160 μL/min. The resulting residue was evaporated under a vacuum to yield pure **3a** as a yellowish solid (891 mg, 2.319 mmol, 98% yield).

### Crystal Structures

Single crystals suitable for X-ray crystallography were obtained by slow evaporation of an acetonitrile solution of **1b**. A suitable crystal was selected and mounted on a SuperNova, Dual, Cu at zero, Atlas diffractometer. The structure was solved with the SHELXT 2014/5^25^ structure solution program and refined with the SHELXL-2018/3^26^ refinement package. Artwork representations were processed using MERCURY^27^ software.

## Supporting Information Available

Syntheses of the resins; reactivation of the basic support; kinetic profiles for the different syntheses; purity comparison for batch vs flow protocols; IR spectra of the distilled solvent; additional experimental setup photos; characterization of the compounds (PDF)
