# Supplementary material for: Continuous Flow Processes as an Enabling Tool for the Synthesis of Constrained Pseudopeptidic Macrocycles
Source: J Org Chem. 2022 Feb 15;87(5):3519–28. doi: 10.1021/acs.joc.1c03081 (PMC9690154; doi:10.1021/acs.joc.1c03081)
Supplement: Supplementary file 1 — jo1c03081_si_001.pdf [file jo1c03081_si_001.pdf]

Continuous flow processes as enabling tool for the synthesis of constrained pseudopeptidic macrocycles

Ferran Esteve,[†] Raul Porcar,[†,‡] Santiago V. Luis[†], Belen Altava,[†]* Eduardo García-Verdugo[†]*

[†] Departamento de Química Inorgánica y Orgánica, Universitat Jaume I, Av. Sos Baynat s/n, 12071, Castellón, Spain.

[‡] Departamento de Química Orgánica y Bio-orgánica, Facultad de Ciencias, Universidad Nacional de Educación a Distancia, UNED, Avda. Esparta, 28232-Las Rozas, Madrid, Spain

Corresponding Author

*E-mail: altava@uji.es.

* E-mail: cepeda@uji.es.

Table of contents

Main discussion SI

Scheme S1. Synthesis of resins S1
Figure S1. IR spectra of freshly prepared, deactivated and reactivated **4** S1
Figure S2. Kinetic profiles for the synthesis of **3a** in batch conditions S2
Figure S3. LHSV vs TOS for the synthesis of **3a** using a cocktail mixture S2
Figure S4. MS-ESI(+) spectra for the batch/flow synthesis of **3b** S3
Figure S5. Kinetic profiles for the synthesis of **3b** in batch/flow conditions S3
Figure S6. IR spectra of distilled and commercially available acetonitrile S4
Figure S7. Optical microscopy of the crystals of **3a** obtained in batch S4
Figure S8. Optical microscopy of the crystals of **3a** obtained in flow S5
Figure S9. ^{1}H NMR spectra for the flow/flow-distillation synthesis of **3a** S5

Experimental part SI S6-S15

MAIN DISCUSSION SI

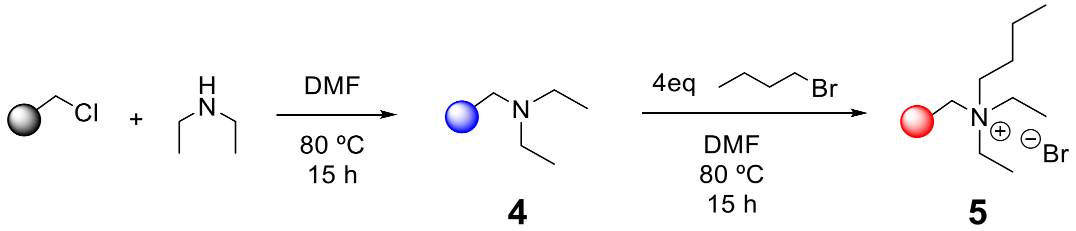

Scheme S1. Synthesis of the polymeric supported base (**4**) and template (**5**). The reactions were performed in an orbital stirrer.

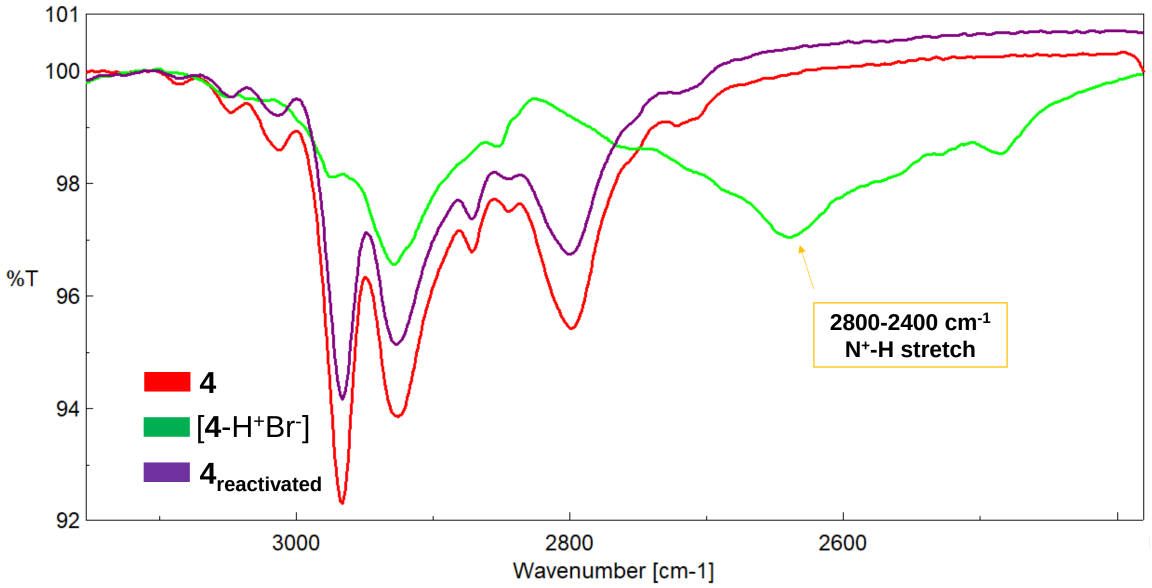

Figure S1. Partial IR spectra of **4** (red), the resin after being filtered-off [**4**-H^+Br^-] (green), and the resin after basic treatment $\mathbf{4}_{\mathbf{reactivated}}$ (purple).

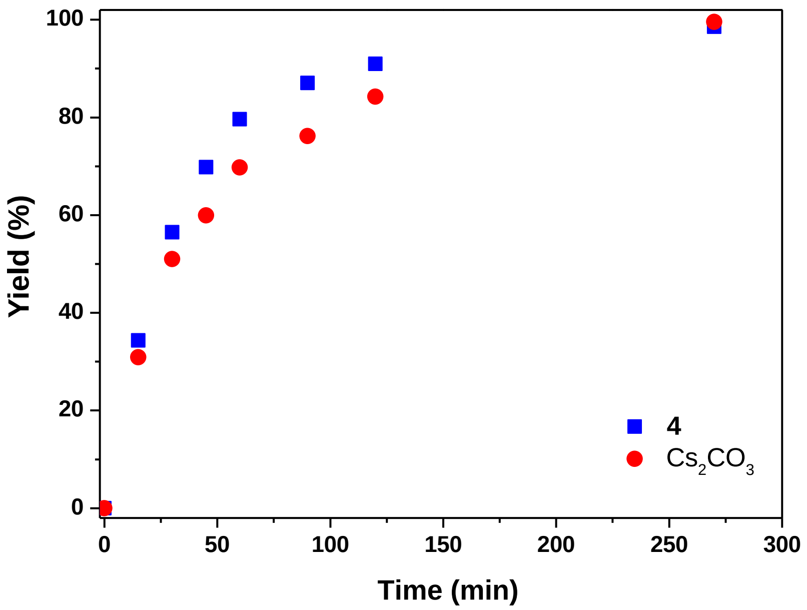

Figure S2. Kinetic profiles when using 6 equivalents of **4** (blue) or caesium carbonate (red) under batch conditions, with a selectivity >99% towards **3a**. Reaction conditions: 80 ºC, 2 mM for **1a** and **2** in CH_3CN. Yields for **3a** were calculated by ^{1}H NMR spectroscopy.

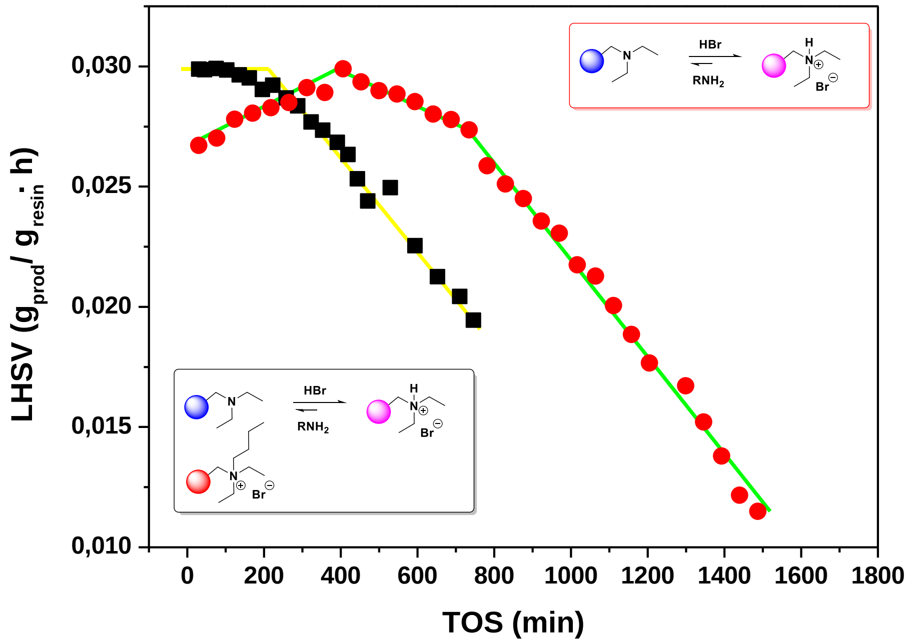

Figure S3. Productivity profiles vs time on stream (TOS). a) Red dots: Supported base **4** (1 g). b) black squares: cocktail mixture of **4** and **5** in a 1:1 molar ratio (1g in total). Conditions: 400 mL/min (2 mM in **1a** and **2**) for 2.2 minutes. LHSV calculated using the yields determined by HPLC, the weight of the polymeric resin and the residence time.

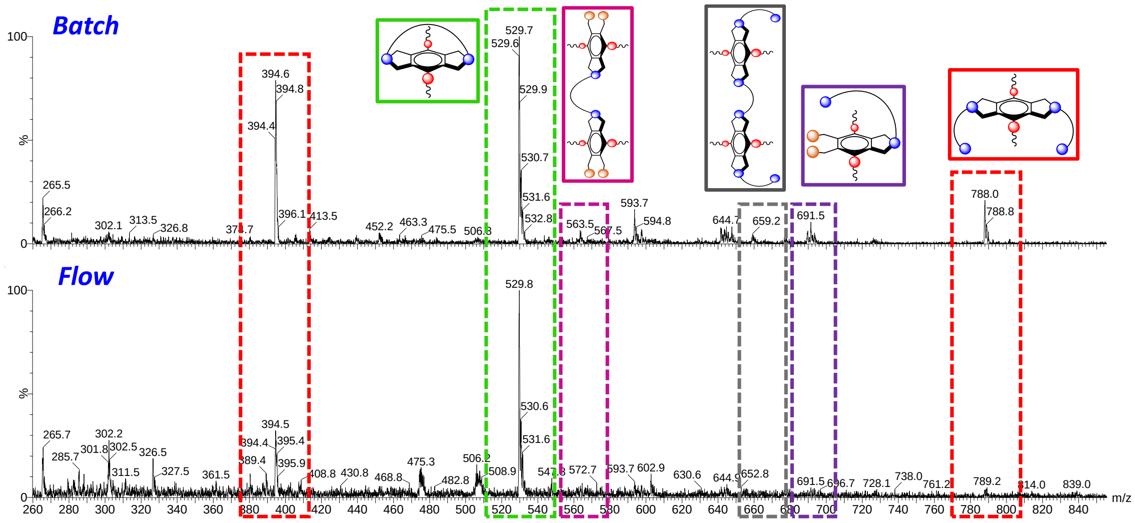

Figure S4. MS-ESI(+) comparison between the reaction crudes obtained under batch conditions (above) and flow conditions (below) using **4** as base.

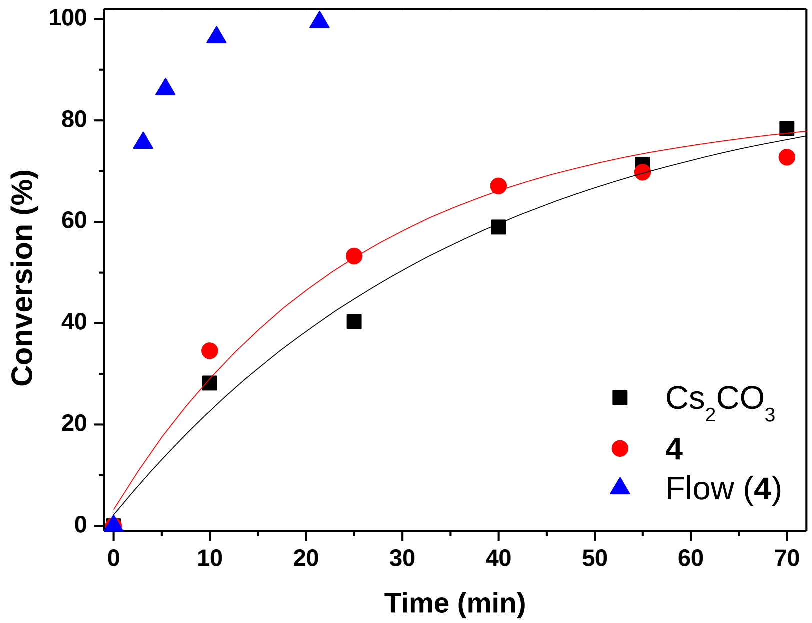

Figure S5. Conversion profiles for **1b** obtained with the different conditions assayed for the synthesis of **3b**. Black: Inorganic base under batch conditions; Red: Basic resin **4** under batch conditions; Blue: Basic resin **4** under flow conditions. All the experiments were carried out with 6 equivalents of the respective base. The blue points correspond to the conversions achieved for the different residence times obtained adjusting the flow. Conversions determined by HPLC. Reaction conditions: 80 ºC, 2 mM in acetonitrile for **1b** and **2**.

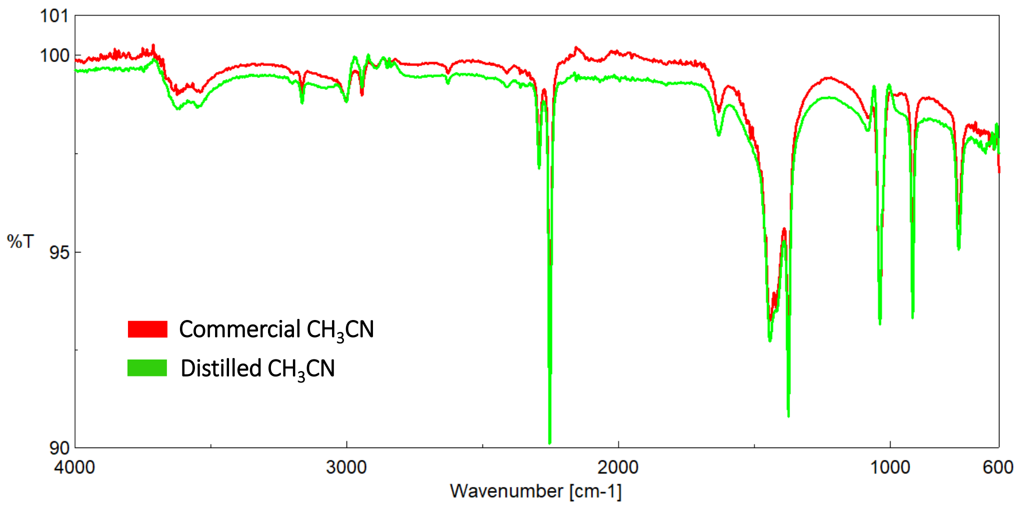

Figure S6. IR spectra comparison between commercial acetonitrile (red spectrum) and the distilled fraction of the flow macrocyclization (green spectrum).

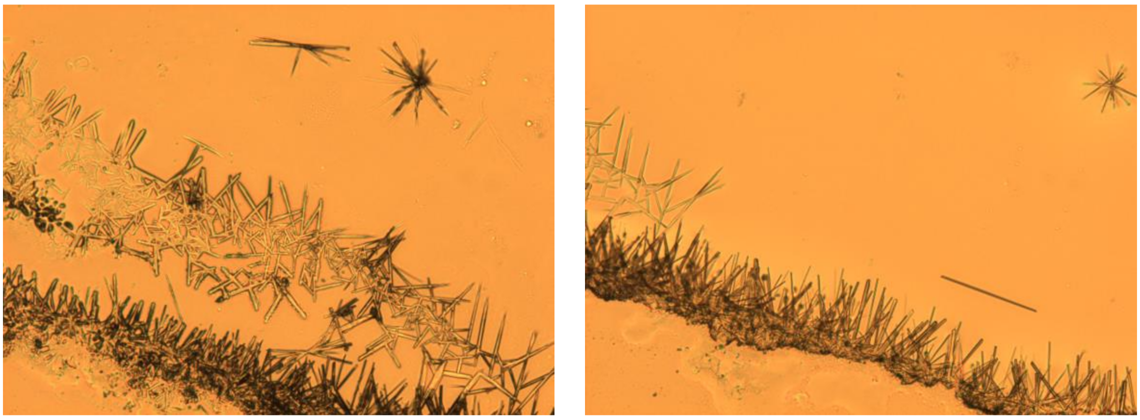

Figure S7. Optical microscopy pictures (40x1) of the **3a** crystals obtained in the reaction crudes after slow evaporation of acetonitrile.

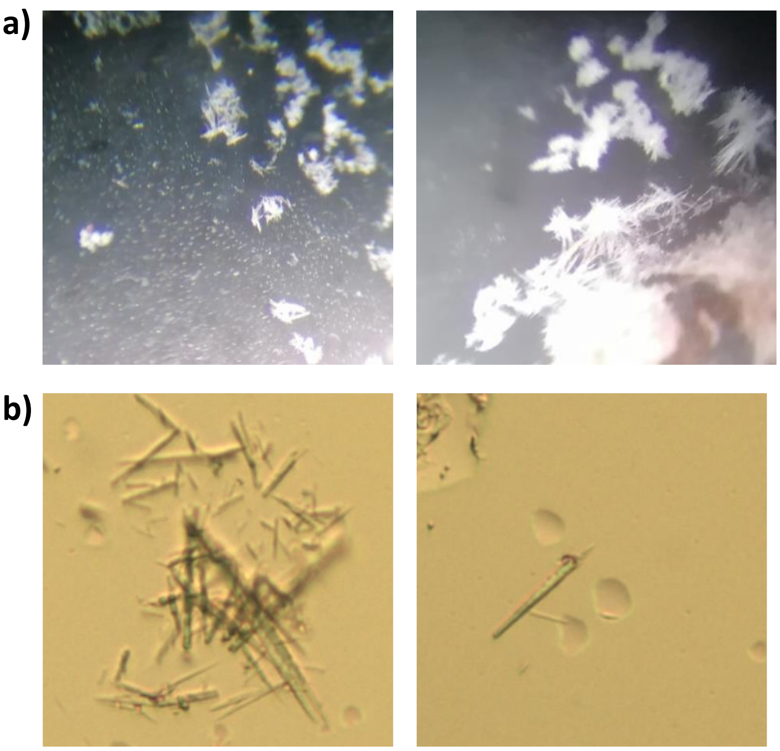

Figure S8. a) Optical microscopy (10x2) pictures of the flow distillation flask walls. b) Optical microscopy pictures (40x2) of the **3a** crystals obtained, extracted from the distillation flask with immersion oil.

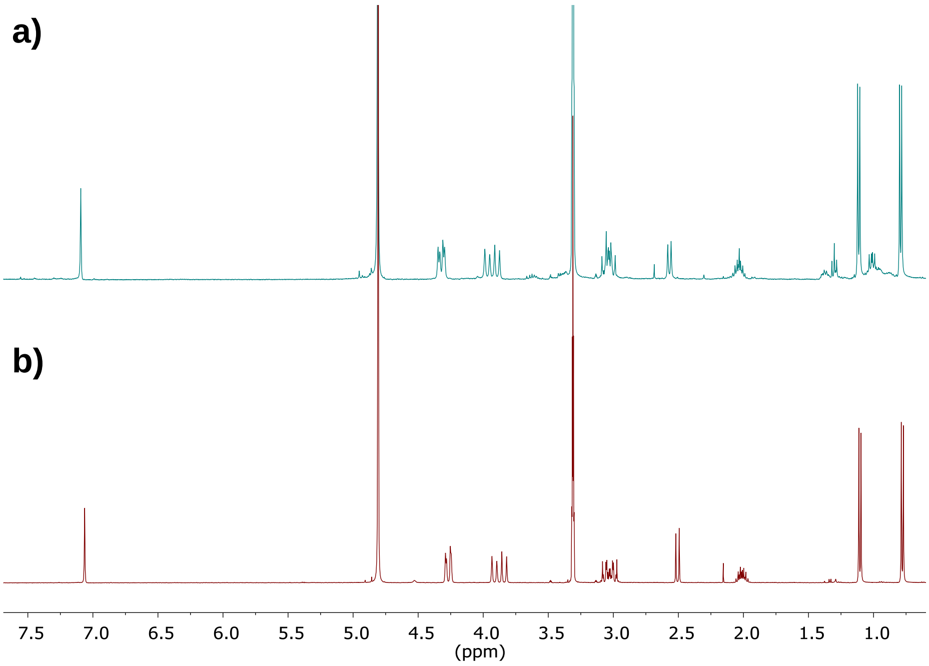

Figure S9. ^{1}H-NMR (400 MHz, 7 mM in CD_3OD) of the reaction crude for a) scaled-up macrocyclization reaction between **1a** and **2**. b) flow-distillation set-up leading to high quality crystals of **3a**.

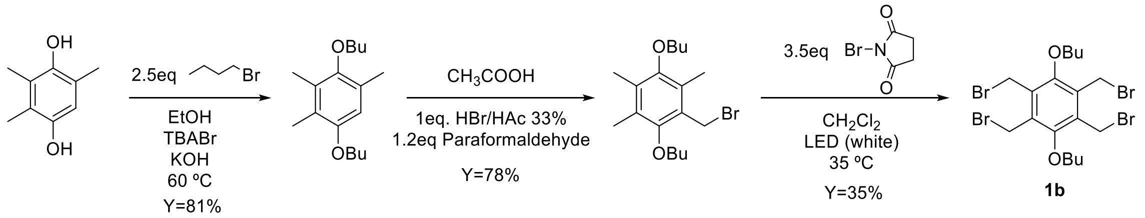

1b

Scheme S2. Synthetic route for **23b**. See reference 2 for more details.

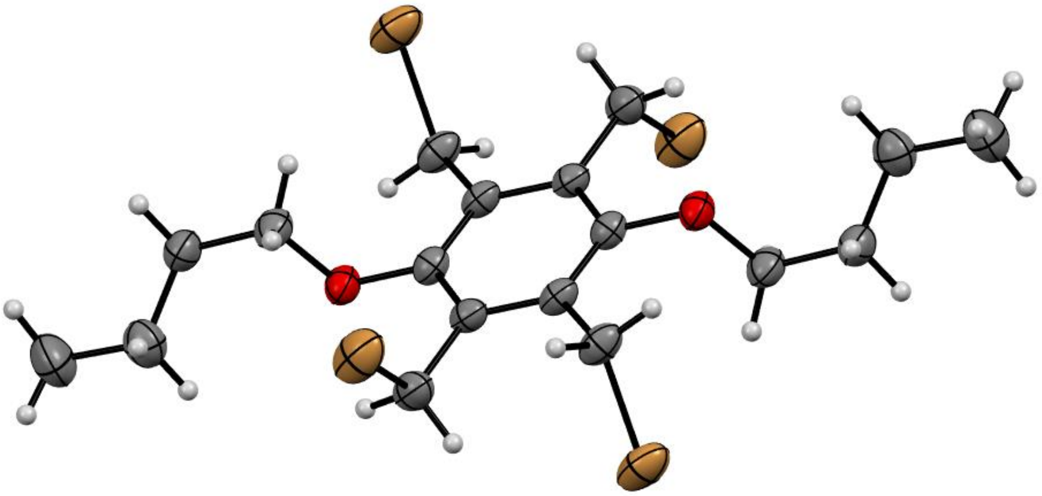

Figure S10. Thermal ellipsoid plot of **1b**. Ellipsoids at 50% of probability.

Table S1. Crystallographic and structural refinement data for compound **1b**.

**Empirical formula**	$C_{18}H_{26}Br_4O_2$
**Formula weight**	594.03
**Temperature/K**	199.9(8)
**Crystal system**	Triclinic
**Space group**	P-1
**a/Å**	10.0246(4)
**b/Å**	10.1498(3)
**c/Å**	11.7979(4)
**α/°**	89.335(3)
**β/°**	70.421(3)
**γ/°**	73.265(3)
**Volume/Å^3**	1078.30(7)
**Z**	2
**ρcalcg/cm^3**	1.830
**F(000)**	580.0
**Crystal size/mm^3**	0.752 × 0.092 × 0.079
**Radiation**	CuKα (λ = 1.54184)
**2Θ range for data collection/°**	7.99 to 143.5
**Index ranges**	$-9 \leq h \leq 12$, $-10 \leq k \leq 12$, $-14 \leq l \leq 14$
**Reflections collected**	11676
**Independent reflections**	4123
**Data/restraints/parameters**	4123/0/229
**Goodness-of-fit on F^2**	1.047
**Final R indexes [I>=2σ (I)]**	R1 = 0.0422, wR2 = 0.1308
**Final R indexes [all data]**	R1 = 0.0468, wR2 = 0.1364
**Largest diff. peak/hole / e Å^{-3}**	0.9/-0.85

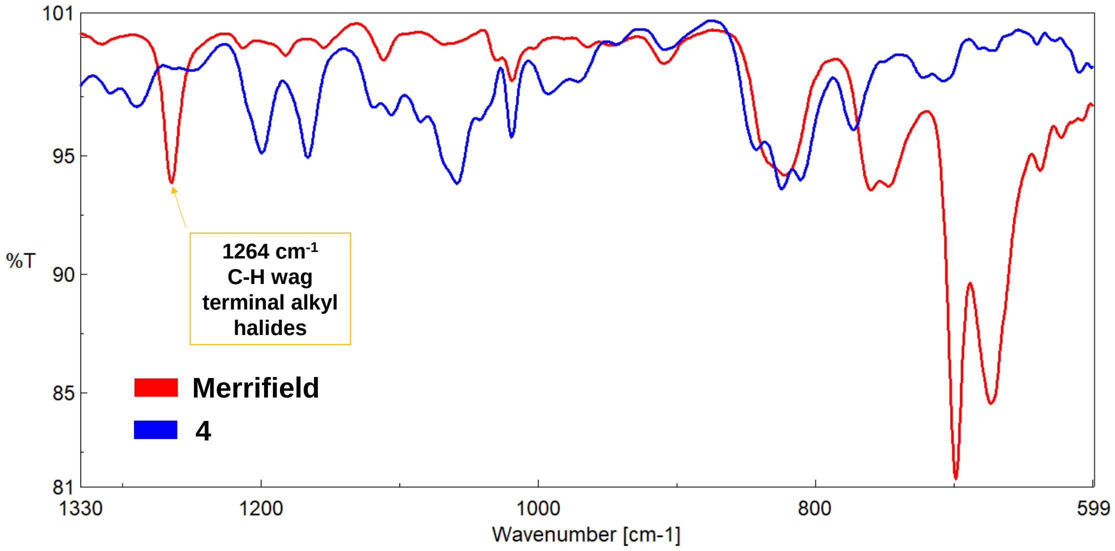

Figure S11. Partial IR spectra for the starting Merrifield resin (5.5 mmol of Cl/g nominal loading) and **4** (blue spectrum). Disappearance of the C-Cl characteristic band has been highlighted in orange.

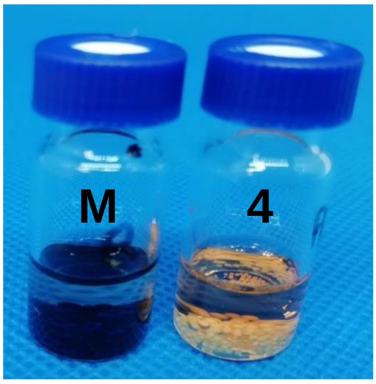

Figure S12. Picture of the NBP test carried out for the supported base **4** (right vial)). The control NBP test for the initial Merrifield (M) resin has also been included (left vial) showing the development of colour associated to the presence of C-Cl groups.

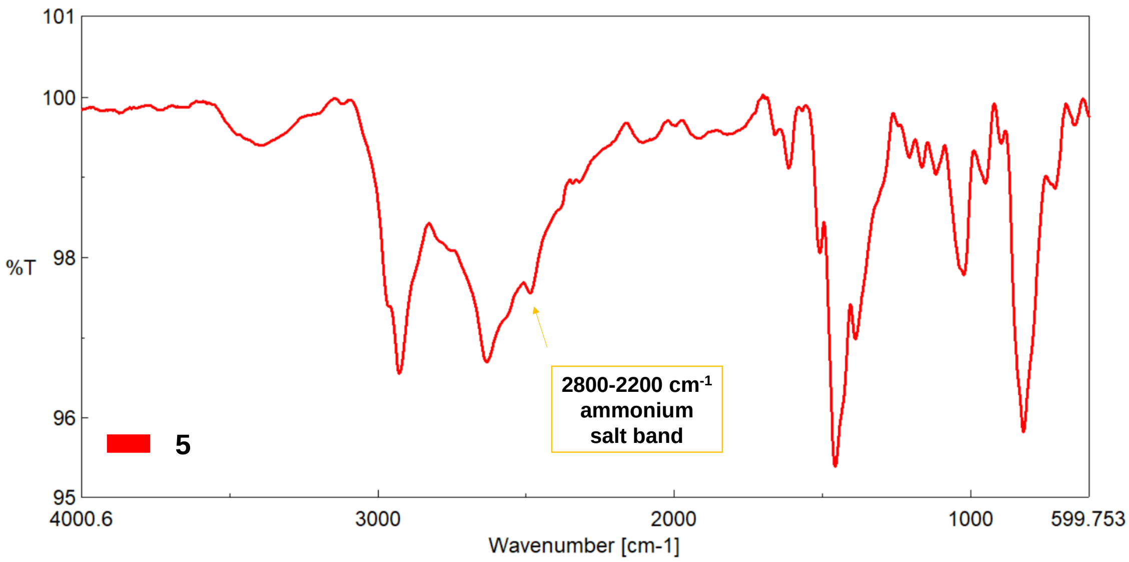

Figure S13. Partial IR spectra for **5**. The presence of a broad band at 2800-2200 cm^{-1} characteristic of ammonium salts has been highlighted in orange.

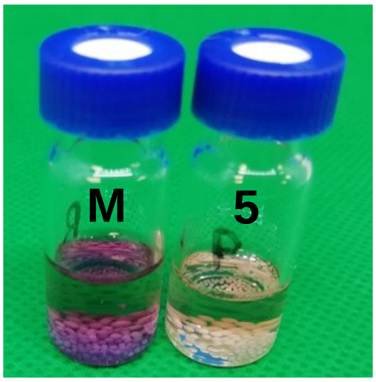

Figure S14. Picture of the NBP test carried out for the supported base **5** (right vial). The control NBP test for the initial Merrifield (M) resin has also been included (left vial).

Table S2. Flash chromatography information for the batch synthesis of **3b**.

**Column**	Silica 40g
**Flow rate**	10 mL/min
**Equilibration Volume**	100.8 mL
**Initial waste**	0 mL
**Air purge**	1 min
**Solvent A**	Dichloromethane
**Solvent B**	Methanol
**Wavelength 1**	254 nm
**Wavelength 2**	220 nm

Table S3. Flash chromatography gradient data for the batch synthesis of **3b**.

**Duration (min)**	**%B (Methanol)**
0	0
15	0
10	3
30	3
30	5
30	5
20	7
2	10
20	10
25	100

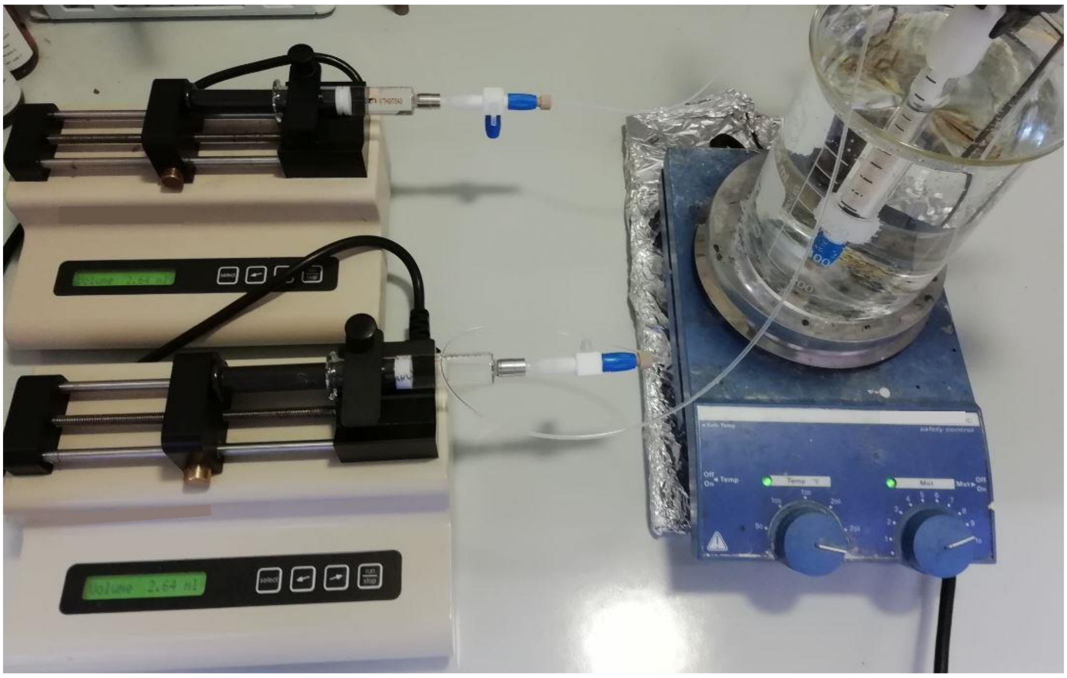

Figure S15. Flow system used for the macrocyclization reaction in CH_3CN, in a water bath at 80 ºC.

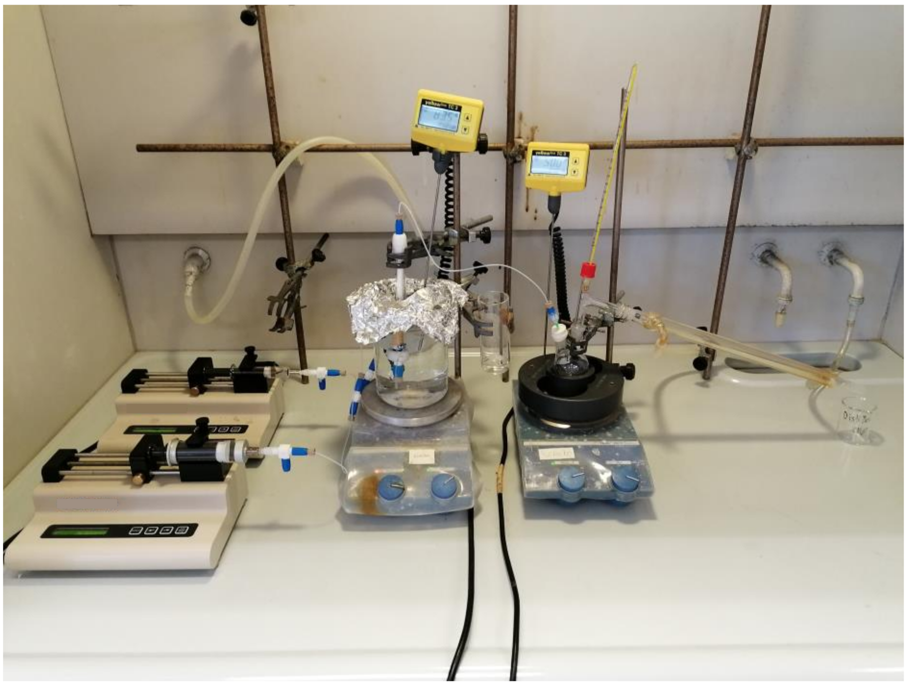

Figure S16. Flow system containing a distillation setup. The recovered acetonitrile was reused to obtain the solutions of the reagents.

Table S4. Flash chromatography information for the flow synthesis of **3b**.

**Column**	Silica 40g
**Flow rate**	10 mL /min
**Equilibration Volume**	81.6 mL
**Initial waste**	0 mL
**Air purge**	1 min
**Solvent A**	Dichloromethane
**Solvent B**	Methanol
**Wavelength 1**	254 nm
**Wavelength 2**	220 nm

Table S5. Flash chromatography gradient data for the flow synthesis of **3b**.

Duration (min)	%B (Methanol)
0	0
5	0
20	3
30	3
30	5
30	5
20	7
17	10
15	20
15	100

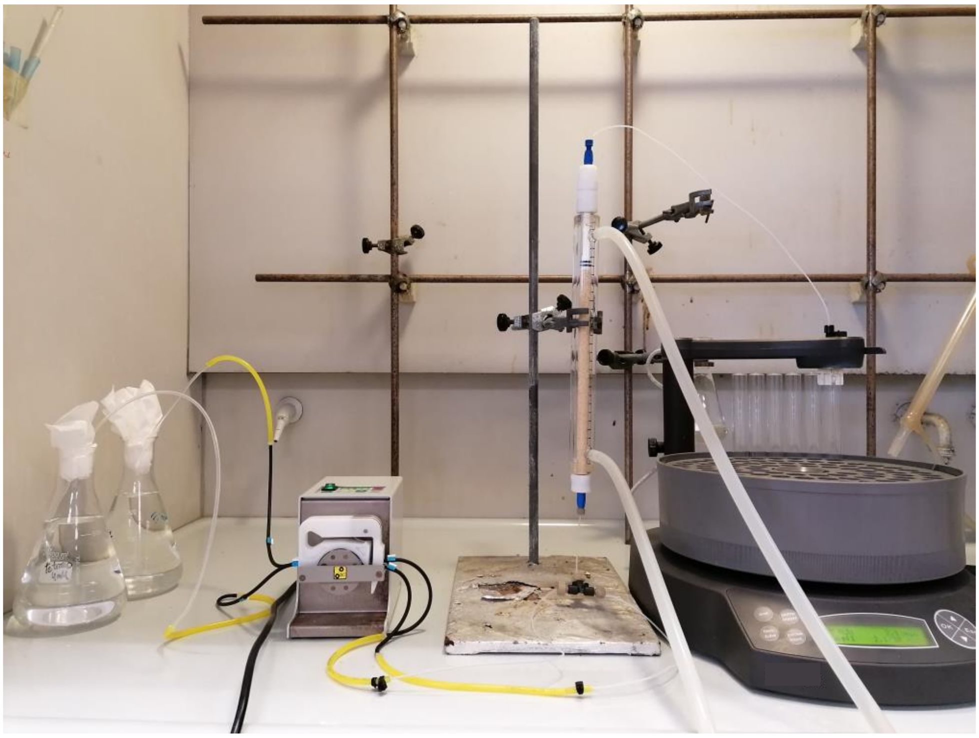

Figure S17. Scaled-up flow setup for the synthesis of **3a** in gram scale.

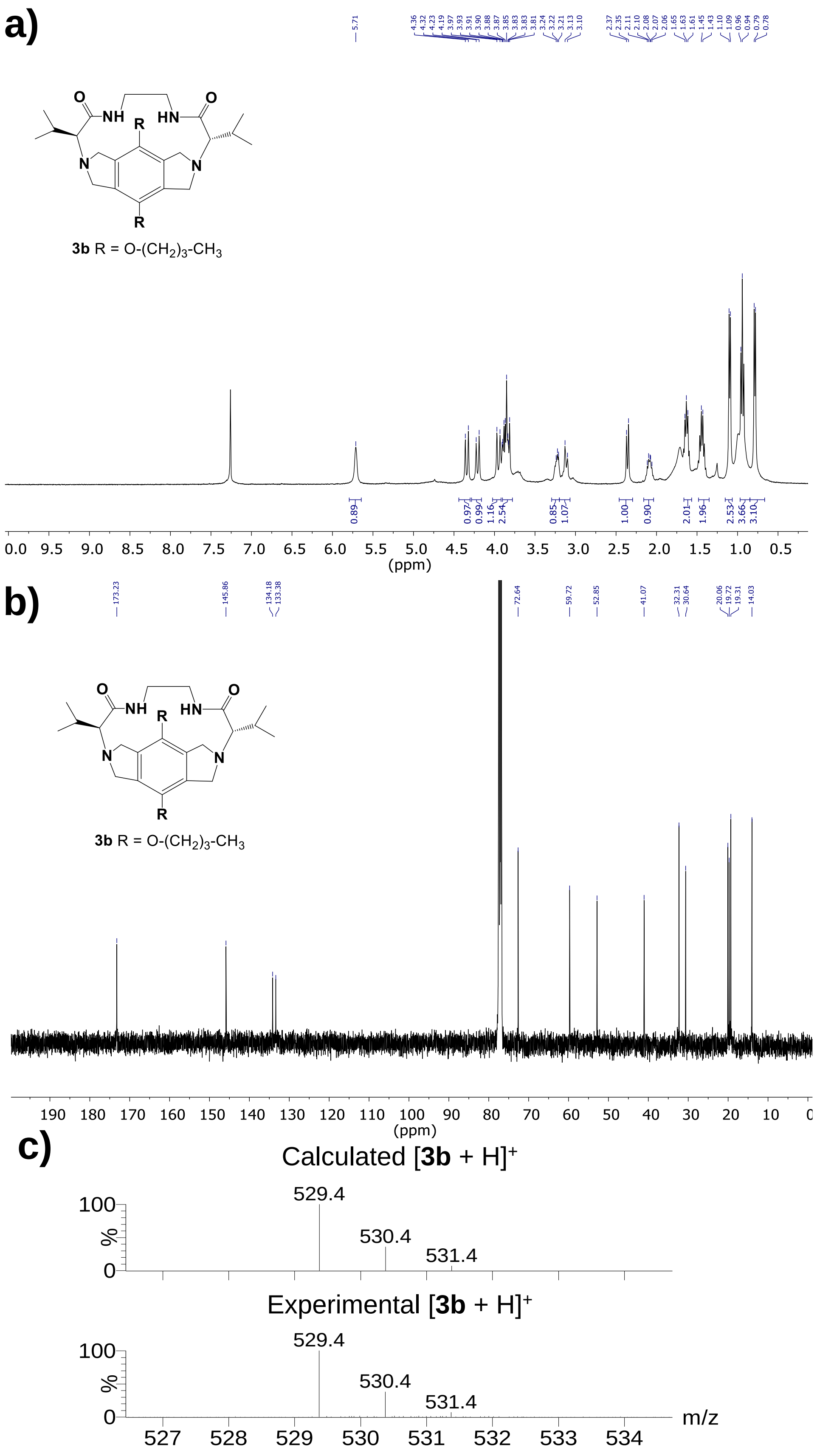

Figure S18. a) ^{1}H-NMR ($CDCl_3$, 400MHz), b) $^{13}C\{^{1}H\}$-NMR ($CDCl_3$, 100MHz) and c) HRMS data for **3b**.

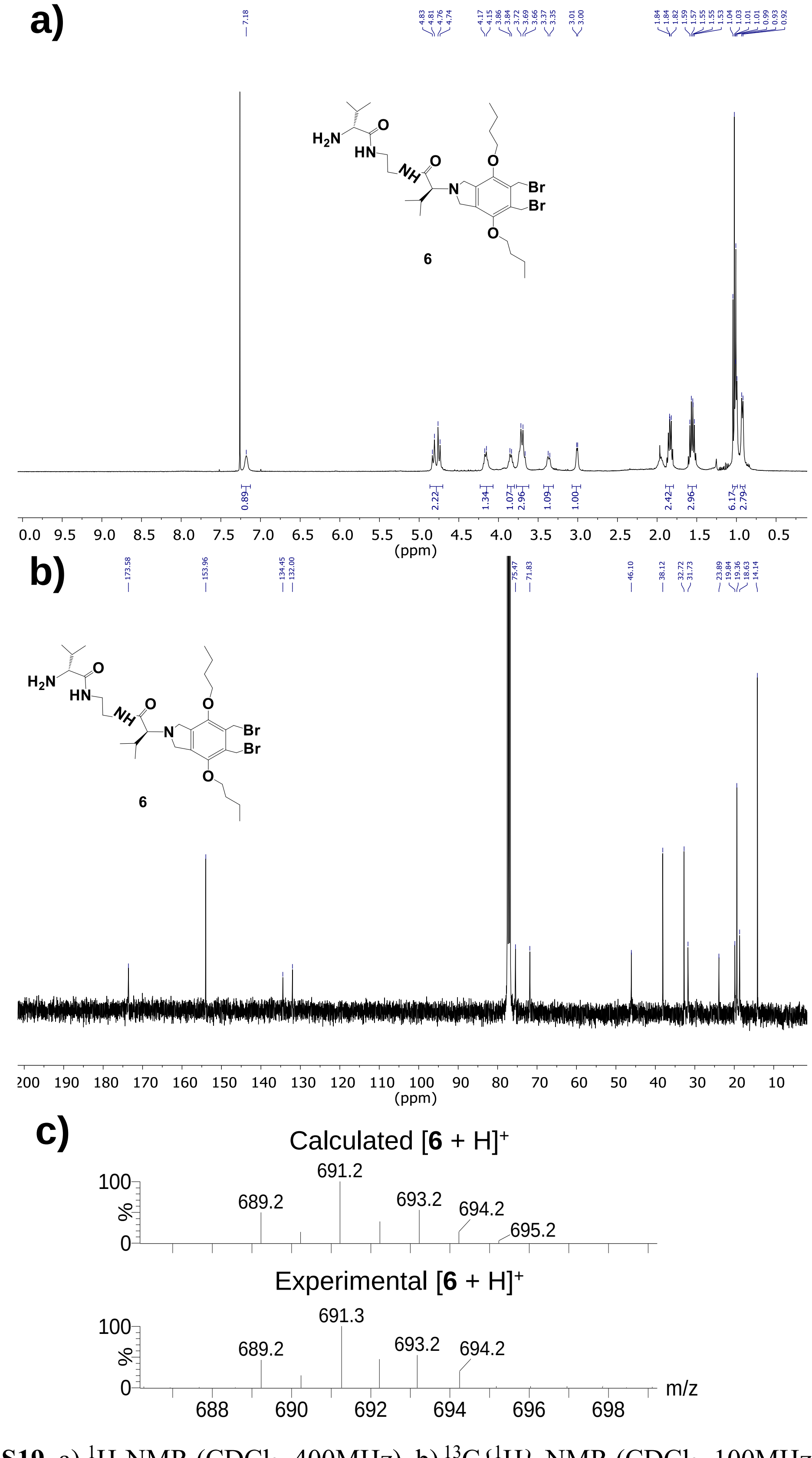

Figure S19. a) ^{1}H-NMR ($CDCl_3$, 400MHz), b) $^{13}C\{^1H\}$-NMR ($CDCl_3$, 100MHz) and c) HRMS data for **6**.

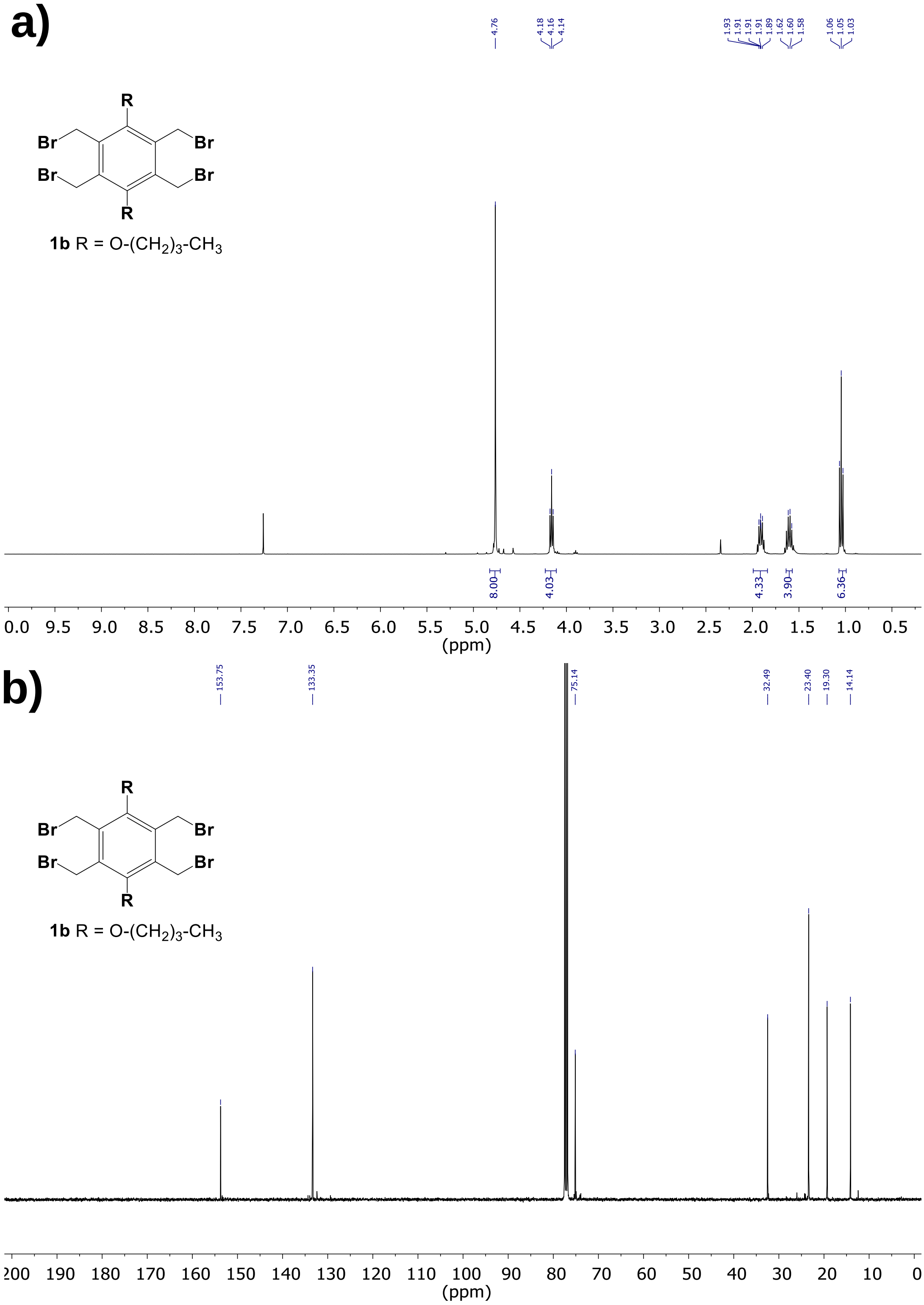

Figure S20. a) ^{1}H-NMR ($CDCl_3$, 400MHz) and b) $^{13}C\{^{1}H\}$-NMR ($CDCl_3$, 400MHz) data for **1b**.

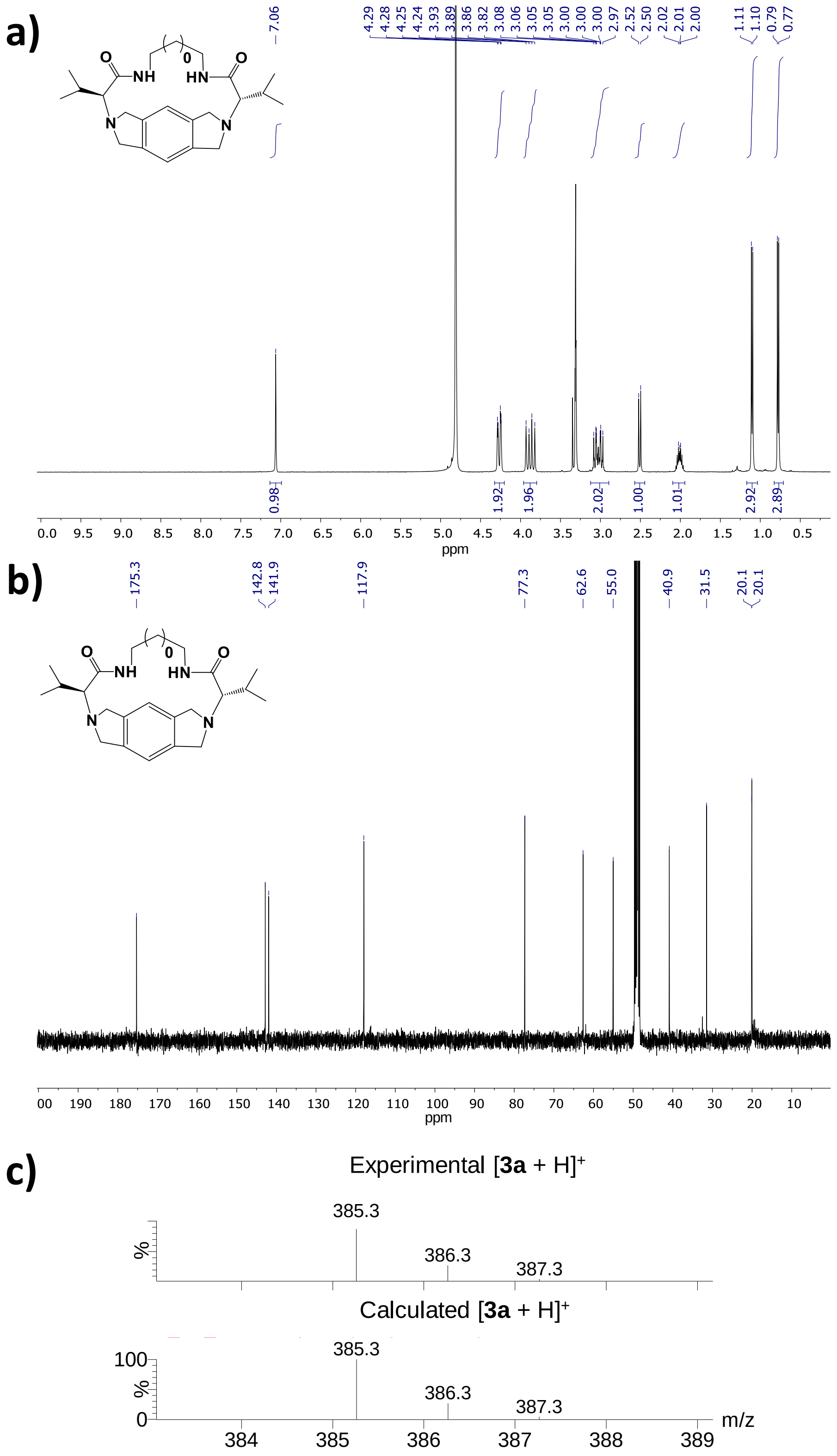

Figure S21. a) ^{1}H-NMR (CD_3OD, 400MHz), b) $^{13}C\{^{1}H\}$-NMR (CD_3OD, 100MHz) and c) HRMS data for **3a**.
